# Concentration with Nanofiltration of Red Wine Cabernet Sauvignon Produced from Conventionally and Ecologically Grown Grapes: Effect on Volatile Compounds and Chemical Composition

**DOI:** 10.3390/membranes11050320

**Published:** 2021-04-27

**Authors:** Ivana Ivić, Mirela Kopjar, Jasmina Obhođaš, Andrija Vinković, Dubravko Pichler, Josip Mesić, Anita Pichler

**Affiliations:** 1 Faculty of Food Technology Osijek, Josip Juraj Strossmayer University, F. Kuhača 18, 31000 Osijek, Croatia; iivic@ptfos.hr (I.I.); mirela.kopjar@ptfos.hr (M.K.); 2 Ruđer Bošković Institute, Bijenička 54, 10000 Zagreb, Croatia; Jasmina.Obhodjas@irb.hr (J.O.); Andrija.Vinkovic@irb.hr (A.V.); 3 Water Supply—Osijek, Poljski Put 1, 31000 Osijek, Croatia; dubravko.pichler@vodovod.com; 4 Polytechnic in Požega, Vukovarska 17, 34000 Požega, Croatia; jmesic@vup.hr

**Keywords:** Cabernet Sauvignon red wine, ecological wine, conventional wine, nanofiltration, volatile compounds, chemical composition, elements concentration

## Abstract

Ecological viticulture represent an upward trend in many countries. Unlike conventional viticulture, it avoids the use of chemical fertilizers and other additives, minimizing the impact of chemicals on the environment and human health. The aim of this study was to investigate the influence of nanofiltration (NF) process on volatiles and chemical composition of conventional and ecological Cabernet Sauvignon red wine. The NF process was conducted on laboratory Alfa Laval LabUnit M20 (De Danske Sukkerfabrikker, Nakskov, Denmark) equipped with six NF M20 membranes in a plate module, at two temperature regimes, with and without cooling and four pressures (2.5, 3.5, 4.5 and 5.5 MPa). Different processing parameters significantly influenced the permeate flux which increased when higher pressure was applied. In initial wines and obtained retentates, volatile compounds, chemical composition and elements concentration were determined. The results showed that the higher pressure and retentate cooling was more favourable for total volatiles retention than lower pressure and higher temperature. Individual compound retention depended on its chemical properties, applied processing parameters and wine composition. Nanofiltration process resulted in lower concentrations of ethanol, acetic acid (>50%), 4-ethylphenol and 4-ethylguaiacol (>90%). Different composition of initial feed (conventional and ecological wine) had an important impact on retention of elements.

## 1. Introduction

Nanofiltration (NF) represents a pressure-driven, low energy and high-efficiency membrane separation technique [1,2,3]. NF membranes are produced from various materials, usually a strong polymer placed on a supporting layer. Such membrane composition enables high selectivity and endurance during the nanofiltration process [4]. Selective membranes split the initial feed into two fractions: the retentate or concentrate that is retained on the membrane and the permeate that passes through it. Comparing to the other membrane filtration methods, nanofiltration falls between ultrafiltration (UF) and reverse osmosis (RO), according to its membrane selectivity and pore size. NF membranes retain smaller molecules than UF ones, but they yield some ions and low molecular weight compounds that are retained on RO membranes [5]. Membrane characteristics are usually expressed through molecular weight cut-off (MWCO) that is the lowest molecular weight retained on the membrane. The MWCO value for NF membranes can vary from 150 to 1000 Da (or g/mol), depending on the membrane type and manufacturer [6]. However, the retention of small molecules results in osmotic pressure increase on the membrane surface and high working pressure should be applied in order to ensure permeate flow, usually between 10 and 30 bar [7]. Higher pressure results in higher permeate flux, faster membrane fouling and concentration polarization [8]. Membrane fouling leads to flux decrease and limits the use of a membrane, but it can also lead to higher desirable compounds retention [9]. These properties enable the application of nanofiltration membranes in various industries: water and wastewater industry, dairy, sugar, beverage, pharmaceutical industries and others [2]. For example, nanofiltration has been globally used for water desalination, cleaning, softening [10,11] or for water pathogens removal [12]. In the dairy industry, salt or acid removal, increasing lactose content and whey protein concentration could be conducted by nanofiltration [6,13]. The ability of NF membranes to retain bioactive compounds, enables the use of those membranes for phenolic compounds recovery from various matrices, for example olive mill wastewater [14]. Further, nanofiltration process can be conducted at room temperatures, ensuring their advantage over thermal processes for concentration. Therefore, it has been widely used for various juices concentrations and correction of juice chemical composition [6,8,15,16].

Membrane filtrations have been used in the wine industry for a long time, for example, microfiltration for clarification of must and wine. However, nanofiltration, along with reverse osmosis, has found its purpose in the wine industry [17]. Water, acetic acid, ethanol and certain low molecular weight compounds and ions can pass through the nanofiltration membranes, but a high percentage of desirable compounds are retained on them. This enables their usage for wine and must concentration, sugar or aroma enhancement, alcohol and acid content correction, flaw elimination (undesirable aroma) and others [18]. Wine dealcoholisation by nanofiltration has become the centre of interest of many studies because this separation technique does not include high-temperature application, it consumes a low amount of energy and minimally changes the initial properties of wine [5,18,19,20,21]. Further, the nanofiltration membranes could be used for polyphenol fractionation and extraction from grape pomace [3,22] or even from wine wastewaters [23], for wine aroma enhancement or correction [24] or acetic acid separation [25,26].

Nanofiltration treatment can be applied for all wine types, regardless of the colour, chemical properties or different production methods. However, each wine matrix is different and this will influence the nanofiltration process and final retentate composition. In this study, Cabernet Sauvignon red wines produced from conventionally and ecologically grown grapes were subjected to the nanofiltration process. The main difference between these two types of viticulture is the application of synthetic nutrients and pesticides. Unlike conventional viticulture, the ecological one avoids the use of chemical additives, fertilizers, pesticides or other products in order to obtain a wine that does not contain any chemical residues [27,28]. This type of viticulture should reduce the impact of chemical products application on the environment and human health [29]. A vineyard where ecological wine will be produced should be certified with precise localisation and date when this type of viticulture began and wine bottles should have an appropriate label [28,30]. However, ecological viticulture does encourage the use of natural or organic additives or adjuvants, for example, systematic fungicides replaced with copper [31]. During harvesting, grapes are not collected by machinery, but exclusively by hand, ensuring reduced damage to the vine, soil and collection of most healthy and ripe grapes [27]. Viticulture methods, environmental factors, berries condition and vinification techniques greatly affect wine chemical composition and aroma [32]. Wine aroma compounds originate from grapes and are formed during the fermentation process or wine storage and aging [24]. Volatile compounds are responsible for wine scent and the final wine aroma will depend on their concentrations and interactions between other wine components (polyphenols, sugars etc.) [33]. Ethanol is the most dominating alcohol in wine and its content should be controlled in order to avoid negative effects on wine quality and aroma [34]. Chemical elements in wine (calcium, iron, copper, zinc, manganese, potassium, bromine, lead and others) have also an important role in wine aroma. Some of them (Zn, Cu) have a beneficial effect on human health in low concentrations. However, excessive concentration of elements, including the ones without nutritional value (lead), affects wine aroma negatively and they are potentially toxic for the human body [35,36].

The aim of this study was to establish the influence of the nanofiltration process under different operating conditions on conventional and ecological Cabernet Sauvignon red wine properties. Different operating conditions included four pressures (2.5, 3.5, 4.5 and 5.5 MPa) and two temperature regimes (with and without retentate cooling). During the nanofiltration process, the retentate temperature and permeate flux were monitored. In obtained retentates, volatile compounds, chemical composition and concentration of elements were determined and compared to the initial wine. In addition, the retention of mentioned compounds in conventional and ecological wine retentates were compared.

## 2. Materials and Methods

### 2.1. Chemical and Standards

In order to conduct this study, myrtenol standard was purchased from Sigma-Aldrich (St. Lois, MO, USA) and sodium chloride was obtained from Kemika (Zagreb, Croatia). Standards of Se, K, Ca, Mn, Fe, Cu, Zn, Br, Rb, Sr and Pb (1 g/L) were purchased from TraceCERT, Fluka Analytical (St. Gallen, Switzerland).

### 2.2. Conventional and Ecological Wine

Conventional and ecological Cabernet Sauvignon red wines (vintage 2018) were produced at cultivation area Zmajevac, Baranja vineyard, Croatia. Conventional grape production included minimally 6 treatments of grapevine with commercial copper-based adjuvants. In rainy seasons, number of treatments could be increased. In ecological viticulture, elementary sulphur and copper (up to 3 kg/ha during one vegetation) were used for 10 treatments of grapevine. Copper was used only until the flowering stage. Any additional treatments included herbal adjuvants with EKO certificate, aminoacids, flavonoids or Neem oil. Ecological wine was treated with minimal amounts of sulphur dioxide in order to prevent wine spoilage. However, maceration of ecological grapes was conducted for several weeks, in order to extract maximal amounts of tannins for more stabilised wine.

### 2.3. Nanofiltration Process

The nanofiltration process was conducted on a laboratory filter with a plate module, LabUnit M20, obtained from De Danske Sukkerfabrikker (Nakskov, Denmark). The plate module was equipped with six composite Alfa Laval NF M20 flat sheet polyamide membranes. These membranes were chosen due to their characteristics: the pH range was from 3 to 10, maximum operating temperature and pressure were 50 °C and 5.5 MPa, respectively, the MgSO_4_ retention (measured on 2000 ppm, 0.9 MPa, 25 °C) was ≥99% and the membrane surface was 0.0289 m^2^. NF M20 membranes showed great selectivity and tolerance to high working pressure and different pH values. In this study, both wines were subjected to a nanofiltration process at four different pressures (2.5, 3.5, 4.5 and 5.5 MPa) and two temperature regimes (with and without retentate cooling). The pressure values were used according to the maximum operating pressure for applied membranes (5.5 MPa) and the difference of 1.0 MPa was applied to obtain the rest of the values. Each experimental run started with 3 L of wine at 15 °C and ended with 1.3 L of retentate and 1.7 L of permeate. During the nanofiltration process, every 4 min the permeate volume and retentate temperature were measured. In order to compare retentates composition with the initial wine, the volume of the permeate that was separated from the wine was replaced with distilled water before each analysis, so the initial wine volume of 3 L was obtained again.

### 2.4. Calculation of Processing Parameters

Permeate flux during the nanofiltration process was calculated by the following formula:

*J* = *V_p_*/(*A* × *t*), (1)

where *J* is permeate flux (L/m^2^h), *V_p_* is permeate volume (L), *A* is membrane surface (m^2^) and *t* is time (hours). Volume reduction factor (*VRF*) was estimated by formula:

*VRF* = *V_f_*/*V_r_*, (2)

where *V_f_* is the initial feed volume (L) and *V_r_* is the retentate volume (L). Water flux was measured before and after each experimental run, in order to calculate fouling index (%):

*FI* = (1 − *J_W_*_1_/*J_W_*_0_) × 100, (3)

where *J_W_*_0_ and *J_W_*_1_ are water fluxes (L/m^2^h) before and after wine concentration, respectively.

### 2.5. Analysis of Volatile Compounds

Volatile compounds in samples were analysed by Agilent 7890B gas chromatograph with Agilent 5977A mass spectrometer (Agilent Technologies, Santa Clara, CA, USA). Sample preparation was conducted with solid-phase microextraction (SPME) where 1 g of sodium chloride was mixed with 5 mL of sample in a 10 mL glass vial. Before extraction, 5 μL of myrtenol (1 mg/L) as an internal standard was added to each sample. Such prepared vials were closed and set on a magnetic stirrer (300 rpm) and heated at 40 °C. In vial headspace, SPME fibre (polydimethylsiloxane/divinylbenzene sorbent, 65 μm, Supelco, Bellefonte, PA, USA) was inserted. After 45 min of adsorption, the SPME fibre was transferred in the GC injection port for 7 min at 250 °C. The volatiles were desorbed at splitless mode into the HP-5MS column (30 m × 0.25 mm × 0.25 μm). The temperature gradient was as follows: from 40 °C (held 10 min) to 120 °C at 3 °C/min; to 250 °C at 10 °C/min. The gas carrier was helium 5.0 (purity 99.999%) and its flow was set at 1 mL/min. Mass spectrometer parameters were the following: MS Source was 230 °C, MS Quad was 150 °C, mass range (*m*/*z*) was 40 to 400 and the ionization energy was 70 eV. Obtained compound peaks were identified according to their mass spectra, retention time and index, NIST (National Institute of Standards and Technology, Gaithersburg, MD, USA) and Wiley mass spectral database. The results were expressed as an average value of three repetitions. For linear retention index calculation purposes, a C7–C30 saturated alkanes standards were analysed under equal conditions.

### 2.6. Analysis of Chemical Composition

The chemical composition of initial ecological and conventional wine and NF retentates was determined on WineScan^TM^ (Foss, Hilleroed, Denmark). Sample was placed in a vial and a sensor was inserted in it. WineScan^TM^ contains FTIR (Fourier Transform Infrared Spectroscopy) interferometer for full infrared scan. The calibration of WineScan^TM^ was conducted with QKit^TM^ 8 (Foss, Hillerød, Denmark).

### 2.7. Determination of Elements (EDXRF Analysis)

Samples of 50 mL were poured into plastic containers (size of 58 × 58 × 40 mm^3^) and 10 µg of Se (TraceCERT 1000 mg/L standard reference material) were added as an internal standard. Afterward, containers were frozen in liquid nitrogen and lyophilized for about 40 h using the Labconco—FreeZone 2.5 L (Labconco Corporation, Kansas City, MO, USA) at −80 °C and pressure of 0.015 mbars. The obtained viscous samples were transferred into plastic holders with the bottom of mylar foil, 3 µm thick. Another layer of mylar foil was carefully glued to the top of the holders to prevent outpouring and contamination of the samples. 

Samples prepared this way were analysed by EDXRF (energy-dispersive X-ray fluorescence) method using Mo anode and Mo secondary target. Working parameters were set to 45 kV and 35 mA and the irradiation time was 1000 s. Samples were irradiated in the air to prevent degassing of volatile components. X-ray spectra were collected using a nitrogen-cooled Canberra Si (Li) detector (Mirion Technologies/Canberra Industries, Meriden, CT, USA) with the active surface of 30 mm^2^, the thickness of 3 mm, Be window thickness of 0.025 mm and FWHM of 170 eV at 5.9 keV. Spectra were analysed using IAEA QXAS software (International Atomic Energy Agency, Seibersdorf, Austria; quantitative X-ray analysis system).

TraceCERT 1000 mg/L certified reference materials were used to create calibration lines for quantification of elements K, Ca, Mn, Fe, Cu, Zn, Br, Rb, Sr and Pb. Relative errors for analysis of elements in wine obtained from the errors of the correlation lines’ coefficients were: K—15.22%, Ca—16.66%, Mn—10.03%, Fe—5.32%, Cu—1.67%, Zn—2.83%, Br—10.82%, Rb—5.34%, Sr—1.98% and Pb—2.74%. MDLs were calculated from the random wine sample by using the equation DL = c*3√ (N_c_)/B, where *c* is the known concentration of the element of interest, *N_c_* is the number of counts under the characteristic X-ray peak and *B* is the number of counts from the background. Calculated MDLs were: 96 mg/L for K; 331 mg/L for Ca; 11 µg/L for Mn; 7 µg/L for Fe; 6 µg/L for Cu; 1.3 µg/L for Zn; 0.823 µg/L for Br; 0.5 µg/L for Rb and Sr; and 0.867 µg/L for Pb. Final concentrations in wine were obtained as the average of triplicate measurements.

### 2.8. Statistical Analysis of Results

All results were expressed as average value and standard deviation. Analysis of variance (ANOVA), Fisher’s least significant difference (LSD) test (*p* < 0.05) and principal component analysis (PCA) were carried out in the statistical software program STATISTICA 13.1 (StatSoft Inc., Tulsa, OK, USA).

## 3. Results

### 3.1. Nanofiltration Processing Parameters

**Figure 1:**
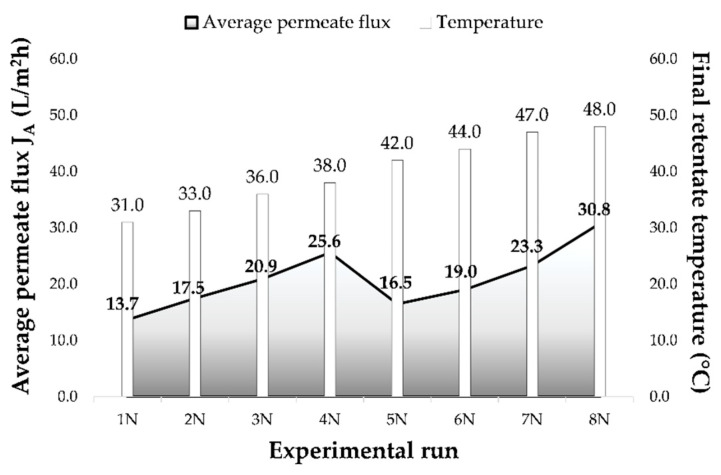
Influence of applied pressure on the average permeate flux *J_A_* (L/m^2^h) and final retentate temperature (°C) during nanofiltration process of conventional and ecological Cabernet Sauvignon red wine. Abbreviations: N—nanofiltration process; 1–2.5 MPa with cooling; 2–3.5 MPa with cooling; 3–4.5 MPa with cooling; 4–5.5 MPa with cooling; 5–2.5 MPa without cooling; 6–3.5 MPa without cooling; 7–4.5 MPa without cooling; 8–5.5 MPa without cooling.

Conventional and ecological Cabernet Sauvignon red wines were concentrated by nanofiltration (NF) at 2.5, 3.5, 4.5 and 5.5 MPa at two temperature regimes. During each process, retentate temperature, volume and time were recorded in order to estimate the influence of applied pressure and temperature regime on permeate flux, final retentate temperature and volume reduction factor (VRF). Retentate temperature at the beginning of each experimental run was 15 °C and it increased during the NF process. Pressure increase resulted in higher retentate temperature (Figure 1) and the highest temperatures were measured in the retentate obtained at 5.5 MPa (38.0 °C with cooling and 48.0 °C without cooling). The cooling regime resulted in about 10 °C lower temperatures than the ones obtained at the regime without cooling. During nanofiltration process, every 4 min permeate volume was measured in order to calculate the permeate flux. Average permeate flux was calculated from obtained fluxes for experimental run. If higher pressure was applied, higher average permeate flux was observed. Therefore, the lowest average permeate flux was measured at 2.5 MPa with cooling (13.7 L/m^2^h) and without cooling (16.5 L/m^2^h). The increase of retentate temperature during the regime without cooling resulted in higher permeate flux than the one obtained when cooling was applied at the same pressure. The highest permeate flux was recorded at 5.5 MPa without cooling (average value was 30.8 L/m^2^h). Same results regarding permeate flux and retentate temperature were obtained for both wines, conventional and ecological.

**Figure 2:**
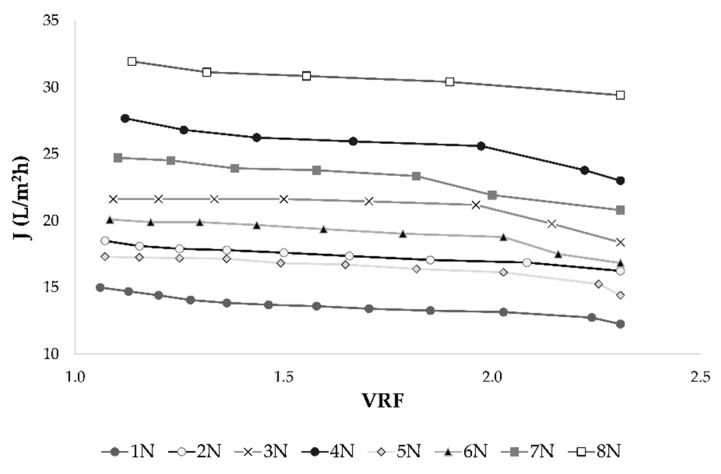
Influence of volume reduction factor (VRF) on permeate flux *J* (L/m^2^h) during nanofiltration process of conventional and ecological Cabernet Sauvignon red wine. Abbreviations: N–nanofiltration process; 1–2.5 MPa with cooling; 2–3.5 MPa with cooling; 3–4.5 MPa with cooling; 4–5.5 MPa with cooling; 5–2.5 MPa without cooling; 6–3.5 MPa without cooling; 7–4.5 MPa without cooling; 8–5.5 MPa without cooling.

Separation of permeate from the initial feed resulted in retentate volume reduction and for each experimental run, VRF was calculated. The VRF value increased as the retentate volume decreased and at the end of each process VRF of 2.31 was achieved. This is accompanied by permeate flux decline (Figure 2), membrane fouling, osmotic pressure increase, concentration polarization and higher retention of most compounds [1].

**Figure 3:**
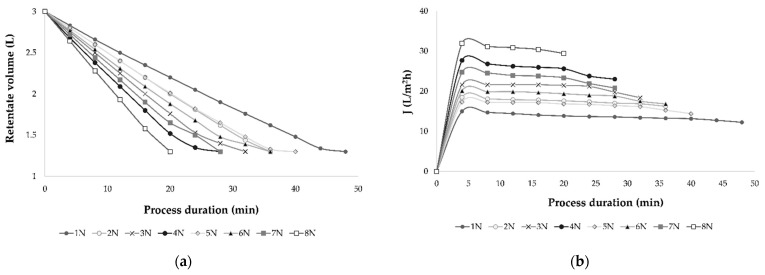
The retentate volume (L) reduction (**a**) and permeate flux *J* (L/m^2^h) decline (**b**) during the nanofiltration process of conventional and ecological Cabernet Sauvignon red wine. Abbreviations: N–nanofiltration process; 1–2.5 MPa with cooling; 2–3.5 MPa with cooling; 3–4.5 MPa with cooling; 4–5.5 MPa with cooling; 5–2.5 MPa without cooling; 6–3.5 MPa without cooling; 7–4.5 MPa without cooling; 8–5.5 MPa without cooling.

Applied pressure influenced permeate flux and membrane fouling. The desired VRF and retentate volume is achieved sooner at higher pressures (4.5 and 5.5 MPa) comparing to the lower pressures (2.5 MPa and 3.5 MPa). The same was observed at processes without cooling that lasted shorter than the ones with cooling at the same transmembrane pressure (Figure 3). The nanofiltration process at 5.5 MPa without cooling resulted in the highest retentate temperature and permeate flux and the VRF value of 2.31 was achieved after only 20 min at those processing parameters. The longest NF process at 2.5 MPa with cooling lasted 48 min but resulted in the lowest retentate temperature.

**Figure 4:**
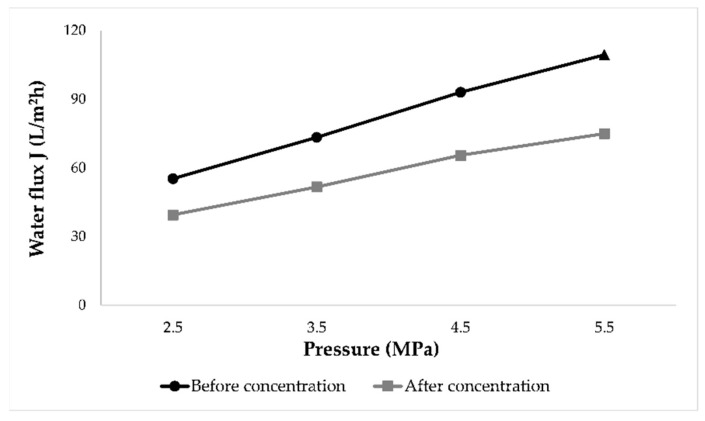
Water fluxes (L/m^2^h) before and after nanofiltration of conventional and ecological Cabernet Sauvignon wine measured on 2.5, 3.5, 4.5 and 5.5 MPa at 25 °C.

In order to determine membrane fouling and flux decline, before and after each wine concentration, pure water flux was measured on 2.5, 3.5, 4.5 and 5.5 MPa. Average values of all experiments were calculated and Figure 4 was obtained. It can be observed that the water flux was lower after wine concentration due to membrane fouling. The decrease of pure water permeability was expressed as fouling index and the results were presented in Table 1. The fouling index ranged from 28.59–31.45%, depending on the applied pressure. Slightly higher fouling index was observed when higher pressure was applied.

### 3.2. Volatile Compounds Retention

Volatile compounds identified in conventional and ecological wine and nanofiltration retentates are presented in Table 2. All volatile compounds were divided into six groups for better display: acids, alcohols, carbonyl compounds, terpenes, esters and volatile phenols. Linear retention index (LRI) was calculated for each volatile compound and the main odour was described.

Concentrations of individual volatile compounds in conventional and ecological Cabernet Sauvignon red wine and nanofiltration retentates are presented in Table 3 and Table 4. All volatile compounds were divided into acids, alcohols, carbonyl compounds, terpenes, esters and volatile phenols and the total sum of each group concentration was calculated. Acetic acid had the highest concentration among six acids (acetic, octanoic, decanoic, lauric, myristic and palmitic acid) identified in initial conventional (394.1 μg/L) and ecological (1043.0 μg/L) red wine. Acetic acid was not detected in any NF retentate regardless of applied pressure or temperature regime. The concentrations of the rest of identified acids decreased after the NF process and the retention of individual acids depended on applied processing parameters.

The cooling regime and higher pressure were more favourable for volatile acid retention. Comparing to the cooling regime, a slight loss was observed when cooling was not applied at the same transmembrane pressure. Lauric acid is an exception; the temperature regime had no significant influence when the pressure of 5.5 MPa was applied in both wine retentates. In ecological wine retentates, the highest concentrations of palmitic acid were estimated at 4.5 and 5.5 MPa with cooling and the lowest ones were measured in the rest of obtained retentates with no significant difference among values regardless of the applied processing parameters. It can be observed that the total concentration of volatile acids decreased in all retentates comparing to the initial conventional (984.1 μg/L) and ecological (1634.4 μg/L) red wine. The highest retention was observed at 5.5 MPa with cooling (41.6% in conventional and 19.6% in ecological wine retentate). However, since NF membranes were permeable for acetic acid and if this acid was not taken into the account, the retention of the total concentration of the rest of volatile acids at 5.5 MPa with cooling was 69.4% in conventional and 54.1% in ecological wine NF retentate. Total concentrations of volatile alcohols in both initial wine and their NF retentates followed the above-mentioned trend: a decrease of total concentration was observed in all obtained retentates comparing to the initial wine, with the highest retention at 5.5 MPa with cooling. In conventional and ecological wine retentates obtained at 5.5 MPa with cooling, 40.3% and 12.9% of total alcohols were retained respectively, comparing to the initial wine. In analysed samples, eight alcohols were identified (isoamyl alcohol; 2,3-butanediol; 1-hexanol; methionol; benzyl alcohol; 1-octanol; 2-phenylethanol; dodecanol), where isoamyl alcohol had the highest concentration in both wines (7.2 mg/L in conventional and 31.8 mg/L in ecological wine). In addition, higher amounts of 2-phenylethanol in initial conventional (4.4 mg/L) and ecological (4.9 mg/L) red wine were detected. The rest of the volatile alcohols had concentrations lower than 1.0 mg/L. In both wine retentates, the highest retention of isoamyl alcohol, 2,3-butanediol, 1-hexanol, 1-octanol and dodecanol was observed at 5.5 MPa with cooling. Lower pressure and higher temperatures (without cooling) resulted in lower retention of mentioned compounds compared to the cooling regime and higher pressures. 

The retention of 2-phenylethanol also increased with lower temperature and higher pressure, but at 5.5 MPa the temperature increase had no significant influence in both wine retentates. Methionol and benzyl alcohol were not detected in NF retentates of ecological Cabernet Sauvignon and in conventional wine retentates, only methionol was detected at 2.5 MPa with cooling (24.0 μg/L). As mentioned before, during the nanofiltration process, a loss of alcohols was observed compared to the initial wine, but the highest retention among all alcohols was observed for dodecanol in conventional and ecological wine retentate (98.1% and 90.7% respectively at 5.5 MPa with cooling).

In both initial wine and nanofiltration retentates, three aldehydes (4-propylbenzaldehyde, lilly aldehyde and hexyl cinnamaldehyde) and one ketone (geranyl acetone) were identified. The total concentration of carbonyl compounds during the NF process decreased comparing to the initial conventional (81.3 μg/L) and ecological (89.4 μg/L) wine. The loss was lower if higher pressure and lower temperature were applied, meaning that the highest total concentration of carbonyl compounds was measured at 5.5 MPa with cooling (57.4 and 59.4 μg/L in conventional and ecological wine retentate, respectively). However, different processing parameters did not affect each compound equally and the retention differed among conventional and ecological wine retentates. In ecological wine retentates, the highest retention of all four carbonyl compounds was observed at 5.5 MPa with cooling. The regime without cooling resulted in lower retention of 4-propylbenzaldehyde and geranyl acetone, comparing to the cooling regime at the same pressures. The temperature regime did not have a significant influence on the retention of lilly aldehyde and hexyl cinnamaldehyde in ecological wine retentates at 2.5, 3.5 and 4.5 MPa. Regarding conventional wine retentates, the lowest concentration of 4-propylbenzaldehyde was measured at 2.5 MPa with cooling (4.3 μg/L), meaning that only 20.3% was retained. The lowest concentration of geranyl acetone among conventional wine retentates was found at 2.5 and 3.5 MPa at both temperature regimes. The pressure increase did not have a significant influence on lilly aldehyde retention in conventional wine retentates when cooling was applied. The highest retention of hexyl cinnamaldehyde was observed at 5.5 MPa with cooling (73.4% was retained comparing to the initial wine).

The retention of five identified terpenes (*α*-terpinolene, *β*-citronellol, *β*-damascenone, *β*-ionone and phenanthrene) during the nanofiltration process of conventional red wine was the lowest at 2.5 MPa without cooling, but it increased with the pressure increment and with retentate cooling. The highest concentrations of α-terpinolene among conventional wine retentates were found at 4.5 and 5.5 MPa with cooling and 5.5 MPa without cooling. In ecological wine, the highest concentration of this compound among retentates was measured at 4.5 MPa with cooling. When cooling was not applied, the pressure changes from 3.5 to 5.5 MPa did not have a significant influence on the retention of *β*-citronellol and *β*-damascenone. On the other hand, higher pressure (5.5 MPa) resulted in higher retention of *β*-citronellol in ecological wine retentates, but the temperature increase when cooling was not applied did not have a significant influence on it comparing to the cooling regime. The behaviour of phenanthrene also differed among retentates of the two wines. In conventional wine retentates, the retention of this compound increased with pressure and lower temperature, with the highest retention achieved at 5.5 MPa with cooling (82.4%) and the lowest one at 2.5 MPa without cooling (39.7%). Further, in ecological wine retentate obtained at 5.5 MPa with cooling, 90.0% of phenanthrene was retained, but the lowest retention was observed at 2.5 and 3.5 MPa at both temperature regimes and 5.5 MPa without cooling (61.4 to 65.7%), with no significant difference among concentrations (*p* > 0.05). The retention of *β*-ionone was the highest at 5.5 MPa with cooling in both wine retentates, but during nanofiltration of conventional wine, the retention of this compound at these processing parameters was higher (56.8%) than the one in ecological wine retentate (20.3%).

Esters were the largest group among all volatiles in analysed samples. Among 19 identified esters, diethyl succinate had the highest concentration in conventional (2.8 mg/L) and ecological (2.9 mg/L) Cabernet Sauvignon wine. Its concentration decreased during nanofiltration treatment of conventional and ecological wine, with the highest concentrations measured at 5.5 MPa with cooling (1.66 and 2.34 mg/L, respectively). The rest of the esters had concentrations lower than 0.5 mg/L. The behaviour of individual esters differed according to the applied processing parameters and type of wine. In global, lower pressure and higher temperature were not favourable for esters retention. This was observed from the total sum of esters concentration in both wine retentates. The highest retention of total esters was detected at 5.5 MPa with cooling (52.2% in conventional and 69.7% in ecological wine retentate). However, the retention of some esters did not follow the mentioned trend. Ethyl hydrogen succinate was only detected in conventional wine retentate at 4.5 and 5.5 MPa with cooling, where 27.9 and 30.1% of initial wine concentration were retained, respectively. Among ecological wine retentates, this compound was found only at 5.5 MPa with cooling (17.5% of initial wine concentration). Similar findings have been established for ethyl linoleate that was only retained in conventional and ecological wine retentate at 5.5 MPa with cooling. Ethyl oleate and ethyl stearate were completely lost during the nanofiltration process of both wines. The rest of the esters were detected in all retentates. For most of them, the retention increased at higher pressure, especially when cooling was applied. However, there are some exceptions. Ethyl vanillate retention in conventional wine retentates increased with higher temperature (regime without cooling) comparing to the cooling regime and the highest concentrations were determined at 3.5, 4.5 and 5.5 MPa without cooling. Ethyl myristate and methyl palmitate concentrations were 16.8 and 7.5 μg/L in initial conventional wine and 13.8 and 14.5 μg/L in ecological Cabernet Sauvignon wine. Their content decreased during the nanofiltration process and the highest retention was observed at 2.5 MPa with cooling for ethyl myristate and 2.5 MPa with and without cooling for methyl palmitate. Pressure increase was not favourable for the retention of these two compounds.

The last group of volatiles in Table 3; Table 4 are volatile phenols where 4-ethylphenol, 4-ethylguaiacol and 2,4-Di-T-butylphenol were located. Their concentration significantly decreased after nanofiltration treatment of conventional and ecological wine, especially the concentrations of 4-ethylphenol and 4-ethylguaiacol. In conventional wine retentates it was retained less than 7% and in ecological wine retentates less than 4% of the initial concentration of 4-ethylphenol and 4-ethylguaiacol. The retention of 2,4-Di-T-butylphenol was 55% or higher in both wine retentates. However, for all volatile phenols, the pressure increases along with retentate cooling resulted in higher retention during the nanofiltration process of conventional and ecological wine comparing to the opposite processing parameters.

**Figure 5:**
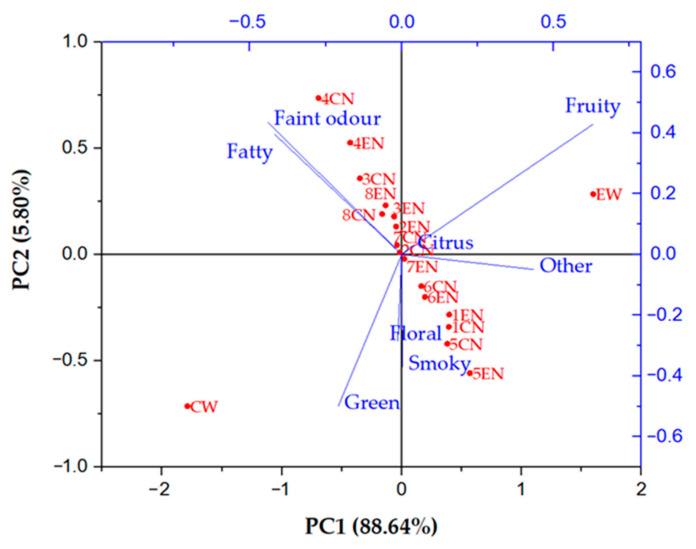
Principal component analysis (PCA) biplot of volatile compounds in initial wines and nanofiltration retentates. Abbreviations: CW—initial conventional wine; EW—initial ecological wine; CN—nanofiltration retentate of conventional wine; EN—nanofiltration retentate of ecological wine; 1–2.5 MPa with cooling; 2–3.5 MPa with cooling; 3–4.5 MPa with cooling; 4–5.5 MPa with cooling; 5–2.5 MPa without cooling; 6–3.5 MPa without cooling; 7–4.5 MPa without cooling; 8–5.5 MPa without cooling.

As mentioned in Table 2, all identified volatile compounds were divided according to their main odour description and eight groups were obtained: fatty, green, floral, citrus, fruity, smoky, faint odour and other (vinegar aroma of acetic acid, sulphurous note of methionol, caramellic note of ethyl 4-hydroxybutanoate and honey aroma of ethyl pentadecanoate). For initial wines and their NF retentates, the total concentration sum of each flavour group was calculated and principal component analysis (PCA) was applied. PCA is a multivariate statistical analysis method that reduces dimensionality and increases interpretability with minimized data loss of large and complex datasets [37]. Figure 5 represents the PCA biplot of analysed wines and nanofiltration retentates. The principal component 1 (PC1), accounting for 88.64% of the total variance, separates the samples on the conventionally produced ones (negative side) and ecologically produced ones (positive side). It is visible that the aroma profiles of the two initial wines are completely different. However, after the nanofiltration process, aroma profiles of both types of retentates have become more similar and the differences were mostly a consequence of different applied pressures or temperature regimes. The PC2, with 5.80% of the total variance, separates the samples according to the applied processing parameters. Conventional and ecological NF retentates obtained at 4.5 and 5.5 MPa with cooling and 5.5 MPa without cooling are clustered on the positive side of PC2. Retentates obtained at 2.5 MPa with cooling and 2.5 and 3.5 without cooling are located on the negative side of PC2. The rest of NF retentates are mostly clustered in the middle of these two.

### 3.3. Chemical Composition of Initial Wine and Nanofiltration Retentates

In initial wines and NF retentates, ethanol, glycerol, density, free and total SO_2_, reducing sugars and CO_2_ were determined. Results are presented in Table 5 and Table 6.

It is visible that initial ethanol content in conventional (13.74 vol.%) and ecological (13.53 vol.%) Cabernet Sauvignon red wine decreased more than 50% after the nanofiltration process. In both wine retentates, the retention of ethanol was similar: slightly higher retention was observed when higher pressure was applied, especially 5.5 MPa, with no significant difference between the two temperature regimes. The retention of glycerol followed a similar trend, with the highest retention estimated at 5.5 MPa with and without cooling. The density slightly increased after nanofiltration treatment in both wine retentates, from 0.9946 g/L in both initial wines to 1.0028 ± 0.0002 g/L in all NF retentates, regardless of the pressure and temperature. Further, the concentration of reducing sugars in both wines was 4.1 g/L and it slightly decreased after nanofiltration of conventional wine comparing to the initial value, with no significant difference among retentates obtained at different operating conditions. However, there was no significant change in reducing sugar content among NF retentates of ecological wine and comparing to the initial value. The highest concentration of CO_2_ was measured in initial conventional and ecological wine (232.61 and 444.64 g/L, respectively. The concentrations decreased after the NF process and higher retention was obtained at lower pressure and temperature, especially at 2.5 MPa with cooling (206.18 g/L in conventional and 160.15 g/L in ecological wine retentate), comparing to the retentates obtained at higher pressures and temperatures. A higher loss of CO_2_ was observed in ecological wine retentates than in the conventional ones, comparing to the initial wines. Both initial wines contained an equal amount of free and total SO_2_ (12.80 and 43.52 g/L, respectively. Free SO_2_ was lower in retentates than in initial wines (at lower pressures 10% and higher pressures 20% lower). Total SO_2_ increased after the NF process, except at 4.5 and 5.5 MPa without cooling in conventional wine retentate (42.24 g/L) and at 5.5 MPa without cooling in ecological wine retentate (43.52 g/L). In ecological wine retentates, higher pressure at cooling was more favourable for total SO_2_ and the opposite was observed in conventional wine retentates. When cooling was not applied, pressure increase lowered the total SO_2_ retention.

In conventional and ecological Cabernet Sauvignon wine and NF retentates, total and volatile acidity, malic, lactic, citric, sorbic and tartaric acid and pH were determined. Obtained results are presented in Table 7 and Table 8.

Results showed that the initial values of total acidity in conventional (4.9 g/L) and ecological (5.1 g/L) wines decreased after NF treatment and there was a significant difference among total acidity of all NF retentates considering pressure and temperature change. The same trend was observed for volatile acids, malic, lactic and citric acid retention. The initial content of these compounds was 0.9, 0.8, 2.1, 0.29 g/L in conventional wine and 0.9, 0.6, 1.8 and 0.31 g/L in ecological wine, respectively. In both wines, the initial content of tartaric acid was 0.7 g/L and it did not significantly change after the nanofiltration process, regardless of the applied pressure and temperature. Further, sorbic acid concentration in initial conventional wine was 132.0 g/L and it significantly decreased during the NF process. The highest retention was measured at 5.5 MPa without cooling (32.0 g/L) followed by 5.5 MPa with cooling (22.0 g/L). Initial ecological wine contained 47.0 g/L of this acid, but it was not detected in any of its NF retentates. The pH was higher in the initial conventional wine (3.92) than in the ecological one (3.75). Those values slightly decreased after NF treatment in all retentates, with no significant change among conventional wine retentates obtained at 2.5, 3.5 and 4.5 MPa with and without cooling, where the lowest values were measured comparing to the retentates at 5.5 MPa at both temperature regimes. In ecological wine retentates, the lowest pH was measured at 2.5 and 3.5 MPa without cooling, comparing to the rest of the retentates, where no significant difference among values was found regardless of the operating conditions.

### 3.4. Elements Content

In conventional and ecological initial wine and NF retentates, 10 elements were determined (potassium, calcium, manganese, iron, copper, zinc, bromine, rubidium, strontium and lead). The results are presented in Table 9 and Table 10.

The results showed that applied pressure and temperature during nanofiltration of conventional and ecological Cabernet Sauvignon did not affect each element equally. In most retentates, a loss of all elements was observed compared to the initial wines. However, in conventional wine retentates, very high retention of K, Ca, Mn, Fe, Cu, Rb and Sr at high pressures was observed, with no significant difference comparing to the concentration of these elements in initial wine. Higher pressure at regime with cooling favoured the retention of potassium, calcium, manganese, iron, bromine, rubidium and strontium in NF retentates of conventional wine. Transmembrane pressure had no significant influence on copper and lead retention, but the temperature increases at the regime without cooling resulted in slightly lower retention of copper, comparing to the cooling regime. The concentration of zinc in initial conventional wine was 1400.5 μg/L. The retention of zinc in conventional wine retentates decreased with higher pressure, meaning that the highest retention was measured at 2.5 MPa with and without cooling (1409.2 and 1380.0 μg/L, respectively). A similar trend was observed in the retention of zinc in ecological wine retentates: the pressure increase resulted in lower retention. Temperature increases at regime without cooling had no significant influence on zinc retention at 3.5, 4.5 and 5.5 MPa, comparing to the cooling regime. The highest concentration of zinc was measured at 2.5 MPa without cooling (1113.4 μg/L, which was 91.8% of the initial concentration). The retention of other elements in ecological wine retentates did not follow the same trend as the ones in conventional wine retentates, for example, pressure and temperature had no significant influence on the retention of potassium and calcium. Pressure increases and retentate cooling favoured the retention of manganese, iron, copper, bromine, rubidium and lead. The highest concentration of strontium in ecological wine retentates was measured at 4.5 MPa with and without cooling (457.0 and 420.8 μg/L, respectively). The concentration of strontium in initial ecological wine was 520.6 μg/L. When cooling was not applied, pressure had no significant influence on the retention of iron, rubidium and lead in ecological wine retentates.

## 4. Discussion

The nanofiltration (NF) process represents a good separation technique that can be applied in various industries, including the wine industry. Selective composite membranes can permeate some ions and low molecular weight compounds, such as water, ethanol, acetic acid and others [18]. The retention of all compounds depends on several factors, such as membrane type and number, membrane material, concentration polarization and fouling, applied transmembrane pressure and retentate temperature, osmotic pressure on the membrane surface, initial feed composition and chemical properties of each compound [8,19,24]. In this study, the influences of four different transmembrane pressures (2.5, 3.5, 4.5 and 5.5 MPa) and two temperature regimes (with and without cooling) were investigated. As expected, the pressure increase resulted in higher permeate flux. Further, the pressure increase led to a higher final retentate temperature, especially when cooling was not applied. The temperature increase resulted in lower retentate viscosity that contributed to the membrane permeability [1,38]. Lower viscosity increases the permeate flux, for example, at the same operating conditions, *n*-hexane flux was higher than water flux due to the lower viscosity of *n*-hexane comparing to water [39]. Higher pressure and permeate flux led to faster wine concentration, but also to sooner membrane fouling, concentration polarization and osmotic pressure increase. This contributed to the retention of most compounds at the beginning of the concentration process, but at a constant pressure, it resulted in permeate flux decline and limited nanofiltration process [8,40]. This was visible from Figure 2, where the influence of volume reduction factor (VRF) on permeate flux was presented. The VRF value increased as the retentate volume decreased during concentration and flux decline is observed. Several studies have obtained similar results [1,5,8,19,41]. Membrane fouling had a great influence on permeate flux and it represents a limiting factor for concentration process. It leads to shorter membrane life, lower productivity, increased investment and difficulties in membrane cleaning [40]. Bartells et al. [42] studied the new generation of low fouling nanofiltration membranes that could minimise the above-mentioned disadvantages. Membrane surface properties affected the membrane fouling mechanism and treatments were required to minimise the interactions between membrane surface and feed components [43]. For example, the effect of photooxidation treatment on nanofiltration membranes was investigated and it was concluded that it decreased the fouling phenomenon [44].

Membrane pore size had a great influence on individual compound retention. Nanofiltration membrane pore sizes are usually around 1 nm [15] and their molecular weight cut-off (MWCO) between 150 and 1000 Da [6]. This means that molecules with molecular weight lower than the MWCO value of the membrane can pass through it. In this study, conventional and ecological Cabernet Sauvignon wines contained the following compounds: water (18.02 g/mol), ethanol (46.07 g/mol), acetic acid (60.05 g/mol), lactic acid (90.08 g/mol), glycerol (92.09 g/mol), malic acid (134.09 g/mol), sorbic acid (112.13 g/mol), tartaric acid (150.09 g/mol), citric acid (192.12 g/mol) and other. In theory, all those compounds should pass through the membrane and the results showed that most of them partially did, but the retention of mentioned compounds during nanofiltration of wine did not depend only on membrane pore size. It also depended on membrane characteristics (material, hydrophobicity, density), applied processing parameters, membrane fouling, wine matrix, chemical properties of individual compounds and chemical interactions between compounds [40]. In retentates obtained in this study, more than 50% of ethanol was removed with NF treatment comparing to the initial wines, meaning that NF membranes can be applied for partial dealcoholisation of wine, as stated in several previous studies [5,20,24]. The viticulture type (conventional or ecological) did not have a significant influence on ethanol retention. Slightly higher retention was only observed when higher pressure was applied, especially at 5.5 MPa at both temperature regimes. However, the retention of other compounds in conventional and ecological wine retentates did not always follow the same trend. For example, reducing sugars did not significantly change during the nanofiltration of ecological wine compared to the initial value, while conventional wine retentates contained slightly lower concentrations of reducing sugars than initial wine. In both wine retentates, a loss of malic, lactic, citric and sorbic acid was determined, but no significant change in tartaric acid content was observed, comparing to the initial wine. Studies showed that nanofiltration membranes, along with reverse osmosis membranes, retain a high percentage of tartaric acid [45] and that they can also be used for tartrate stabilisation [46,47]. Further, the volatile acids content in both wines was decreased after the nanofiltration process due to the separation of acetic acid that is representative of this group. Acetic acid is a secondary product of alcoholic and lactic fermentation, but excessive amounts of this acid lead to wine spoilage and undesirable vinegar aroma [48]. Therefore, the nanofiltration process proved to be good for acetic acid correction, as stated in our previous study [24]. However, Temido et al. [45] have stated that during acetic acid correction it was important to select a membrane that can separate this acid from other organic acids, but the acetic acid and ethanol separation was more difficult and the highest separation was achieved at pH 3.2. The free SO_2_ concentration decreased after the NF process comparing to the initial conventional and ecological wine, but the total SO_2_ concentration increased. The free SO_2_ may have bound with the wine components and the total SO_2_ increased. Sulphur dioxide usually interacts with acetaldehyde, pyruvic acid or α-ketoglutaric acid [49]. However, processing parameters did not affect the content of total SO_2_ equally in both analysed wines. In conventional wine retentates, pressure increase resulted in lower SO_2_ content comparing to the retentates obtained at lower pressures, while in ecological wine retentates the cooling regime resulted in higher SO_2_ concentrations at 3.5, 4.5 and 5.5 MPa.

Elements in wine influence the organoleptic properties of wine. The content of elements in wine depends on viticulture methods, soil characteristics and additives that were used (fungicides, fertilizers or pesticides that contain Cu, Mn, Pb or others) [35]. The nanofiltration process of conventional and ecological wine affected the elements content and high rejection was observed for most of the elements. The retention of all elements depended on applied processing parameters and the wine type. Moreira et al. [50] stated that nanofiltration membranes rejected high concentrations of strontium. In this study, the highest retention of strontium among ecological wine retentates was 87.8% and in conventional wine retentates at 4.5 and 5.5 MPa with cooling, there was no significant change in strontium concentration, comparing to the initial wine. This property of NF membranes is usually used for water treatment and harmful elements removal. Pino et al. [51] stated that the rejection of elements depended on membrane type and operating conditions and Mullett et al. [52] reported that the pH of feed and membrane electrical charge influenced elements and ion retention during nanofiltration of mine water. The pH of the feed influenced the membrane surface charge, permeate flux and retention of individual compounds. Polyamide membranes have an isoelectric point at pH 3.5–4.0, at which maximum permeate flux and the highest permeability occurred [53]. If the pH is higher than the isoelectric point, a negative charge increase will occur on the membrane surface increasing ion rejection [54]. The wine pH is usually in the above-mentioned range. In this study, the pH values of initial conventional and ecological wine were 3.92 and 3.75, respectively. A slight decrease of initial pH value occurred after nanofiltration treatment, but it was still near the isoelectric point of the NF membranes, which could explain the high permeability of certain compounds.

In all analysed samples, 45 volatile compounds, divided into acids, alcohols, terpenes, carbonyl compounds, esters and volatile phenols, were identified and monitored. In addition to acetic acid, five other organic acids (octanoic, decanoic, lauric, myristic and palmitic acid) were identified in conventional and ecological Cabernet Sauvignon wine and NF retentates. A loss of mentioned volatile acids was observed, but the retention depended on applied operating conditions and the highest retention of total acids was achieved at 5.5 MPa with cooling. The retention differed between two types of wine retentates, conventional and ecological. For example, in conventional wine retentates at 5.5 MPa with cooling, 65.8% and 72.2% of initial concentration of octanoic and decanoic acid were measured, respectively. Those values in ecological wine retentates were 24.2% and 92.2%. Similar behaviour was also observed among other volatiles in retentates. In initial wines, the highest concentrations among all groups were measured for alcohols due to the high concentration of isoamyl alcohol and 2-phenylethanol. Higher alcohols contribute to the wine aroma unless their concentration is over 400 mg/L [55]. However, nanofiltration treatment resulted in a significant loss of total alcohols. The highest retention of total alcohols was measured at 5.5 MPa with cooling. The permeability or retention of volatiles did not depend only on applied pressure and temperature, but also on individual compound chemical properties, ability to bind with other compounds, their vapour pressure and volatility [56].

In global, higher pressure and retentate cooling were more favourable for the retention of total volatile compounds than lower pressures and higher temperatures. However, this behaviour was not noticed for each compound. For example, the temperature increase at the regime without cooling did not have a significant effect on β-citronellol retention and the retention of ethyl myristate and methyl palmitate decreased with higher pressure. Esters were the largest group among volatiles, accounting for 19 compounds. In both wines, 30 to 60% of esters were lost during nanofiltration, depending on the applied pressure and temperature regime. Diban et al. [57] reported that esters concentration decreased during partial removal of alcohol in wine due to their hydrophobicity. The hydrophobicity and polarity of NF membranes influenced the retention of individual compounds. Polar membranes showed higher rejection towards nonpolar compounds increasing their retention. If a compound shows a hydrophobic character, it would be attracted to the hydrophobic part of a membrane and this would increase the permeability of this compound, for example, hexanol [58]. In this study, the concentrations of hexanol in retentates were more than 90% lower than in the initial conventional and ecological wine.

The results showed that a certain loss of volatiles occurred during the nanofiltration of Cabernet Sauvignon red wine. However, this can be used for undesirable volatile compounds removals, such as 4-ethylphenol (122.2 g/mol) and 4-ethylguaiacol (152.2 g/mol). In both wines, over 94% of these compounds were removed and in the retentates obtained at 2.5 MPa with cooling, they were not detected. These compounds are produced by *Brettanomyces* yeast and they contribute to the smoky, stable, medicinal or horse sweat aroma. They indicate wine spoilage if present in higher concentrations, as a consequence of inadequate wine storage, especially in wooden barrels [59]. Although previous studies [60,61] showed that reverse osmosis could be used for 4-ethylphenol and 4-ethylguaiacol removal, the results in this study and our previous study [24] showed that nanofiltration membranes are also permeable for these compounds.

As mentioned, except for operating conditions and membrane characteristics, the retention of a compounds during nanofiltration depended on several factors, including their chemical properties and affinity to interact with other compounds in order to increase its stability. Those interactions usually include hydrogen bonding with polyphenols in wine [62]. Therefore, different wine matrix and polyphenol profiles in conventional and ecological wine played a great role in volatiles retention. According to the principal component analysis (PCA), the aroma profile of conventional wine was significantly different from the aroma profile of ecological wine. After the nanofiltration process, the aroma profiles of conventional and ecological wine retentates were more similar, with slight differences regarding the applied pressure and temperature.

## 5. Conclusions

Nanofiltration proved to be applicable for wine concentration, partial dealcoholisation or chemical composition correction. Although a loss of certain desirable compounds occurred (volatile compounds, lactic, malic, citric and sorbic acid, glycerol and most elements), the nanofiltration process still has several advantages over thermal concentration processes: low energy consumption, high efficiency and selectivity and minimal degradation of initial feed components due to mild temperatures. The results in this study showed that operating conditions, such as transmembrane pressure and retentate temperature, significantly affected the permeate flux and compounds retention. Higher pressure and resulted in higher permeate flux, higher retentate temperature and faster wine concentration, but also in higher membrane fouling comparing to lower pressures. The retention of compounds depended on many factors, including the chemical composition of the initial feed. Results showed that the retention of individual compounds differed among conventional and ecological retentates at the same operating conditions. In both wine retentates, retention of total volatile compounds was higher when cooling and higher pressure were applied, unlike opposite conditions. The retention of total acids, alcohols and terpenes was higher in conventional than in ecological wine retentates, where higher retention of total carbonyl compounds and esters was measured. Nanofiltration resulted in a change of aromatic profile of both wines. Applied nanofiltration membranes were permeable for ethanol, acetic acid and undesirable aroma compounds, 4-ethylphenol and 4-ethylguaiacol that makes them applicable for wine chemical composition correction. The retention of elements depended on applied processing parameters that influenced each element differently.

## Figures and Tables

**Table 1:** Fouling index (%) of nanofiltration membranes at four different pressures.

Pressure (MPa)	Fouling Index (%)
2.5	28.59
3.5	29.54
4.5	29.60
5.5	31.45

**Table 2:** Volatile compounds, their linear retention indices (LRI) and odour description in conventional and ecological Cabernet Sauvignon red wine and retentates obtained by nanofiltration at 2.5, 3.5, 4.5 and 5.5 MPa with and without cooling.

Compound	LRI	Odour	Compound	LRI	Odour
Acids			Carbonyl compounds
Acetic acid	622	vinegar	4-propylbenzaldehyde	1261	faint
Octanoic acid	1199	fatty	Geranyl acetone	1448	floral
Decanoic acid	1376	fatty	Lily aldehyde	1517	floral
Lauric acid	1556	fatty	Hexyl cinnamaldehyde	1738	floral
Myristic acid	1749	fatty	Terpenes		
Palmitic acid	2004	fatty	*α*-terpinolene	1092	citrus
Alcohols			*β*-citronellol	1223	citrus
Isoamyl alcohol	734	fruity	*β*-damascenone	1377	fruity
2,3-butanediol	804	fruity	*β*-ionone	1476	fruity
1-hexanol	868	green	Phenanthrene	1772	faint
Methionol	981	sulphurous	Volatile phenols		
Benzyl alcohol	1037	fruity	4-ethylphenol	1166	smoky
1-octanol	1071	green	4-ethylguaiacol	1268	smoky
2-phenylethanol	1103	floral	2,4-Di-T-butylphenol	1501	faint
Dodecanol	1469	fatty			
Esters					
Ethyl hexanoate	997	fruity	Ethyl myristate	1778	fatty
Ethyl 4-hydroxybutanoate	1060	caramellic	Diisobutyl phthalate	1859	faint
Diethyl succinate	1179	fruity	Ethyl pentadecanoate	1880	honey
Ethyl octanoate	1191	fruity	Methyl palmitate	1907	fatty
Ethyl hydrogen succinate	1198	faint	Dibutyl phthalate	1953	faint
Phenethyl acetate	1248	floral	Ethyl palmitate	1978	fatty
Ethyl decanoate	1391	fruity	Ethyl linoleate	2146	fatty
Ethyl vanillate	1580	smoky	Ethyl oleate	2152	fatty
Ethyl laurate	1584	fatty	Ethyl stearate	2176	fatty
Hexyl salicylate	1667	green			

**Table 3:** Volatile compounds identified in conventional Cabernet Sauvignon red wine and nanofiltration retentates at 2.5, 3.5, 4.5 and 5.5 MPa with cooling and without cooling. Different superscript letters (a, b, c, d, e, f, g, h, i) in the same row represent statistical difference by ANOVA, Fisher’s (LSD) test (*p* < 0.05).

Compound	CW	1CN	2CN	3CN	4CN	5CN	6CN	7CN	8CN
∑Acids (μg/L)	984.1 ± 10.8 ^i^	252.5 ± 4.2 ^b^	338.7 ± 5.5 ^e^	371.9 ± 8.1 ^g^	409.4 ± 4.8 ^h^	225.0 ± 2.8 ^a^	287.1 ± 4.2 ^c^	320.1 ± 5.9 ^d^	353.6 ± 5.9 ^f^
Acetic acid (μg/L)	394.1 ± 3.2 ^a^	-	-	-	-	-	-	-	-
Octanoic acid (μg/L)	341.6 ± 5.3 ^h^	136.9 ± 0.9 ^b^	187.1 ± 2.5 ^e^	205.5 ± 5.1 ^f^	224.9 ± 2.3 ^g^	106.8 ± 0.5 ^a^	154.2 ± 2.3 ^c^	167.1 ± 2.2 ^d^	183.2 ± 2.2 ^e^
Decanoic acid (μg/L)	172.4 ± 1.5 ^f^	85.1 ± 2.3 ^a^	104.8 ± 2.2 ^c^	112.6 ± 1.7 ^d^	124.5 ± 0.9 ^e^	85.0 ± 2.1 ^a^	99.8 ± 0.7 ^b^	106.9 ± 3.1 ^c^	114.3 ± 2.4 ^d^
Lauric acid (μg/L)	45.7 ± 0.1 ^g^	16.5 ± 0.5 ^a^	32.4 ± 0.4 ^d^	35.8 ± 0.9 ^e^	37.5 ± 0.9 ^f^	21.1 ± 0.1 ^b^	20.7 ± 1.0 ^b^	30.9 ± 0.4 ^c^	38.6 ± 1.2 ^f^
Myristic acid (μg/L)	22.0 ± 0.7 ^f^	12.2 ± 0.5 ^b^	12.0 ± 0.3 ^b^	14.2 ± 0.2 ^c^	18.1 ± 0.6 ^d^	10.6 ± 0.2 ^a^	10.6 ± 0.1 ^a^	12.1 ± 0.1 ^b^	14.1 ± 0.1 ^c^
Palmitic acid (μg/L)	8.3 ± 0.1 ^g^	1.9 ± 0.1 ^b^	2.5 ± 0.1 ^c^	3.8 ± 0.2 ^e^	4.3 ± 0.1 ^f^	1.5 ± 0.1 ^a^	1.9 ± 0.1 ^b^	3.1 ± 0.1 ^d^	3.5 ± 0.1 ^e^
∑Alcohols (mg/L)	13.21 ± 0.06 ^h^	3.83 ± 0.09 ^b^	4.08 ± 0.07 ^d^	4.63 ± 0.05 ^f^	5.32 ± 0.05 ^g^	2.70 ± 0.05 ^a^	3.97 ± 0.06 ^c^	4.07 ± 0.07 ^d^	4.36 ± 0.04 ^e^
Isoamyl alcohol (mg/L)	7.15 ± 0.02 ^h^	2.38 ± 0.06 ^b^	2.74 ± 0.05 ^d^	3.19 ± 0.04 ^f^	3.77 ± 0.02 ^g^	1.59 ± 0.01 ^a^	2.61 ± 0.04 ^c^	2.68 ± 0.05 ^cd^	2.89 ± 0.01 ^e^
2,3-butanediol (μg/L)	507.2 ± 0.8 ^g^	12.1 ± 0.1 ^b^	12.6 ± 0.5 ^b^	29.6 ± 0.3 ^d^	57.3 ± 0.1 ^f^	-	11.5 ± 0.2 ^a^	20.7 ± 0.2 ^c^	42.7 ± 0.8 ^e^
1-hexanol (μg/L)	868.4 ± 8.0 ^h^	52.4 ± 0.1 ^c^	70.2 ± 0.4 ^e^	77.8 ± 1.2 ^f^	87.0 ± 1.3 ^g^	44.9 ± 0.4 ^a^	51.1 ± 0.9 ^b^	51.6 ± 0.1 ^b^	53.0 ± 0.4 ^d^
Methionol (μg/L)	45.9 ± 1.2 ^b^	24.0 ± 0.3 ^a^	-	-	-	-	-	-	-
Benzyl alcohol (μg/L)	48.6 ± 0.1 ^a^	-	-	-	-	-	-	-	-
1-octanol (μg/L)	57.0 ± 0.1 ^g^	35.9 ± 0.5 ^d^	36.2 ± 0.4 ^d^	37.7 ± 0.3 ^e^	42.1 ± 1.0 ^f^	31.3 ± 0.2 ^a^	32.8 ± 0.1 ^c^	31.6 ± 0.3 ^b^	35.8 ± 0.8 ^d^
2-phenylethanol (mg/L)	4.42 ± 0.03 ^d^	1.15 ± 0.04 ^b^	1.14 ± 0.03 ^b^	1.20 ± 0.03 ^bc^	1.26 ± 0.03 ^c^	0.98 ± 0.03 ^a^	1.18 ± 0.02 ^b^	1.19 ± 0.02 ^b^	1.25 ± 0.03 ^c^
Dodecanol (μg/L)	113.8 ± 1.7 ^f^	66.6 ± 0.6 ^b^	89.3 ± 3.0 ^d^	94.1 ± 1.2 ^e^	111.6 ± 1.1 ^f^	58.5 ± 0.8 ^a^	74.9 ± 1.2 ^c^	91.1 ± 1.0 ^d^	93.4 ± 0.1 ^e^
∑Carbonyl compounds (μg/L)	81.3 ± 2.0 ^h^	38.1 ± 1.1 ^b^	46.2 ± 2.1 ^de^	49.5 ± 0.6 ^f^	57.4 ± 1.1 ^g^	35.5 ± 0.8 ^a^	40.7 ± 1.2 ^c^	45.1 ± 1.0 ^d^	48.5 ± 0.9 ^ef^
4-propylbenzaldehyde (μg/L)	21.2 ± 0.6 ^h^	4.3 ± 0.1 ^a^	12.4 ± 0.7 ^de^	13.1 ± 0.2 ^e^	17.7 ± 0.1 ^g^	8.5 ± 0.1 ^b^	10.5 ± 0.1 ^c^	11.5 ± 0.5 ^d^	15.3 ± 0.1 ^f^
Geranyl acetone (μg/L)	24.4 ± 0.2 ^d^	13.7 ± 0.4 ^a^	14.1 ± 0.6 ^a^	15.2 ± 0.2 ^b^	17.0 ± 0.3 ^c^	13.3 ± 0.2 ^a^	13.3 ± 0.4 ^a^	15.2 ± 0.2 ^b^	15.8 ± 0.4 ^b^
Lily aldehyde (μg/L)	19.9 ± 1.1 ^e^	11.4 ± 0.6 ^d^	10.3 ± 0.5 ^d^	10.5 ± 0.2 ^d^	11.0 ± 0.4 ^d^	6.5 ± 0.4 ^a^	8.3 ± 0.3 ^b^	9.5 ± 0.1 ^c^	8.7 ± 0.1 ^b^
Hexyl cinnamaldehyde (μg/L)	15.8 ± 0.1 ^f^	8.6 ± 0.1 ^b^	9.5 ± 0.3 ^c^	10.6 ± 0.1 ^d^	11.6 ± 0.3 ^e^	7.2 ± 0.2 ^a^	8.6 ± 0.4 ^b^	9.0 ± 0.2 ^c^	8.8 ± 0.3 ^b^
∑Terpenes (μg/L)	194.4 ± 5.0 ^f^	71.7 ± 1.6 ^b^	75.3 ± 1.5 ^c^	85.1 ± 2.3 ^d^	92.3 ± 1.2 ^e^	56.3 ± 1.4 ^a^	70.7 ± 1.9 ^b^	75.7 ± 1.1 ^c^	83.6 ± 1.1 ^d^
*α*-terpinolene (μg/L)	87.3 ± 2.9 ^d^	32.2 ± 1.2 ^b^	32.9 ± 1.1 ^b^	37.3 ± 1.3 ^c^	37.0 ± 0.4 ^c^	26.7 ± 1.0 ^a^	32.0 ± 0.8 ^b^	32.3 ± 0.2 ^b^	36.8 ± 0.2 ^c^
*β*-citronellol (μg/L)	20.6 ± 0.2 ^f^	11.5 ± 0.1 ^c^	12.2 ± 0.1 ^d^	12.7 ± 0.2 ^e^	13.1 ± 0.2 ^e^	9.1 ± 0.1 ^a^	10.4 ± 0.4 ^b^	10.4 ± 0.2 ^b^	10.5 ± 0.1 ^b^
*β*-damascenone (μg/L)	48.0 ± 0.8 ^f^	15.6 ± 0.1 ^d^	14.8 ± 0.1 ^c^	15.0 ± 0.2 ^c^	18.6 ± 0.1 ^e^	9.9 ± 0.1 ^a^	13.3 ± 0.3 ^b^	13.2 ± 0.4 ^b^	13.9 ± 0.5 ^b^
*β*-ionone (μg/L)	31.7 ± 1.1 ^f^	8.6 ± 0.1 ^b^	11.4 ± 0.3 ^c^	16.1 ± 0.5 ^d^	18.0 ± 0.2 ^e^	7.8 ± 0.3 ^a^	11.5 ± 0.3 ^c^	15.8 ± 0.1 ^d^	17.6 ± 0.2 ^e^
Phenanthrene (μg/L)	6.8 ± 0.1 ^f^	3.7 ± 0.1 ^b^	4.0 ± 0.1 ^c^	4.1 ± 0.1 ^c^	5.6 ± 0.3 ^e^	2.7 ± 0.1 ^a^	3.5 ± 0.1 ^b^	3.9 ± 0.2 ^bc^	4.8 ± 0.1 ^d^
∑Esters (mg/L)	4.08 ± 0.05 ^g^	1.79 ± 0.02 ^c^	1.82 ± 0.06 ^cd^	1.93 ± 0.04 ^e^	2.13 ± 0.02 ^f^	1.63 ± 0.03 ^a^	1.75 ± 0.03 ^bc^	1.71 ± 0.04 ^b^	1.89 ± 0.01 ^de^
Ethyl hexanoate (μg/L)	156.8 ± 1.5 ^e^	47.6 ± 0.7 ^c^	47.8 ± 0.6 ^c^	49.0 ± 1.0 ^c^	55.2 ± 0.2 ^d^	40.8 ± 0.9 ^a^	45.6 ± 0.5 ^b^	48.8 ± 1.1 ^c^	49.5 ± 1.3 ^c^
Ethyl 4-hydroxybutanoate (μg/L)	53.5 ± 0.1 ^c^	12.2 ± 0.2 ^a^	11.9 ± 0.7 ^a^	12.8 ± 0.3 ^a^	18.0 ± 0.1 ^b^	12.7 ± 0.5 ^a^	12.9 ± 0.3 ^a^	12.7 ± 0.1 ^a^	12.2 ± 0.2 ^a^
Diethyl succinate (mg/L)	2.84 ± 0.04 ^e^	1.51 ± 0.01 ^b^	1.52 ± 0.05 ^b^	1.52 ± 0.03 ^b^	1.66 ± 0.01 ^d^	1.38 ± 0.02 ^a^	1.48 ± 0.02 ^b^	1.44 ± 0.04 ^ab^	1.60 ± 0.01 ^c^
Ethyl octanoate (μg/L)	346.7 ± 1.6 ^h^	85.2 ± 0.5 ^d^	95.1 ± 0.6 ^e^	99.3 ± 2.7 ^f^	112.1 ± 5.9 ^g^	62.0 ± 0.5 ^a^	70.5 ± 0.1 ^b^	71.0 ± 0.7 ^bc^	71.9 ± 0.9 ^c^
Ethyl hydrogen succinate (μg/L)	282.3 ± 8.7 ^c^	-	-	79.0 ± 0.3 ^a^	85.1 ± 0.8 ^b^	-	-	-	-
Phenethyl acetate (μg/L)	64.2 ± 2.5 ^f^	41.6 ± 1.3 ^a^	42.0 ± 0.6 ^a^	48.4 ± 0.1 ^c^	56.0 ± 2.0 ^e^	41.4 ± 0.4 ^a^	42.3 ± 0.3 ^a^	46.3 ± 0.1 ^b^	52.7 ± 0.2 ^d^
Ethyl decanoate (μg/L)	26.1 ± 1.4 ^d^	8.6 ± 0.4 ^b^	9.5 ± 0.5 ^b^	9.4 ± 0.9 ^b^	10.8 ± 0.2 ^c^	5.2 ± 0.1 ^a^	8.9 ± 0.2 ^b^	9.4 ± 0.4 ^b^	10.4 ± 0.1 ^c^
Ethyl vanillate (μg/L)	10.7 ± 0.3 ^f^	4.7 ± 0.3 ^a^	5.9 ± 0.6 ^b^	7.4 ± 0.1 ^d^	6.9 ± 0.1 ^c^	5.9 ± 0.2 ^b^	8.5 ± 0.1 ^e^	8.4 ± 0.1 ^e^	8.5 ± 0.1 ^e^
Ethyl laurate (μg/L)	23.3 ± 1.2 ^f^	10.5 ± 0.1 ^b^	10.9 ± 0.6 ^b^	15.6 ± 0.4 ^d^	19.9 ± 0.1 ^e^	9.8 ± 0.3 ^a^	9.4 ± 0.1 ^a^	10.6 ± 0.2 ^b^	12.0 ± 0.3 ^c^
Hexyl salicylate (μg/L)	18.5 ± 0.5 ^e^	10.1 ± 0.2 ^b^	11.2 ± 0.2 ^c^	13.5 ± 1.3 ^d^	17.1 ± 0.9 ^e^	7.4 ± 0.1 ^a^	10.5 ± 0.1 ^b^	10.6 ± 0.1 ^b^	11.1 ± 0.1 ^c^
Ethyl myristate (μg/L)	16.8 ± 0.2 ^g^	9.0 ± 0.1 ^f^	6.1 ± 0.1 ^d^	6.2 ± 0.3 ^d^	6.2 ± 0.2 ^d^	6.6 ± 0.1 ^e^	5.0 ± 0.1 ^c^	3.6 ± 0.1 ^b^	2.5 ± 0.1 ^a^
Diisobutyl phthalate (μg/L)	34.4 ± 0.3 ^h^	18.0 ± 0.1 ^b^	19.4 ± 0.1 ^c^	20.5 ± 0.4 ^d^	26.4 ± 0.1 ^g^	17.4 ± 0.2 ^a^	18.6 ± 0.2 ^b^	21.9 ± 0.1 ^e^	25.4 ± 0.2 ^f^
Ethyl pentadecanoate (μg/L)	15.7 ± 0.1 ^g^	6.3 ± 0.1 ^a^	7.0 ± 0.1 ^b^	8.8 ± 0.1 ^d^	13.6 ± 0.6 ^f^	13.5 ± 0.3 ^f^	10.4 ± 0.1 ^e^	7.5 ± 0.2 ^c^	7.5 ± 0.2 ^c^
Methyl palmitate (μg/L)	7.5 ± 0.2 ^e^	4.6 ± 0.2 ^d^	3.1 ± 0.1 ^b^	2.8 ± 0.2 ^b^	2.2 ± 0.2 ^a^	4.7 ± 0.7 ^d^	3.9 ± 0.1 ^c^	3.6 ± 0.3 ^c^	3.0 ± 0.2 ^b^
Dibutyl phthalate (μg/L)	33.3 ± 0.4 ^g^	12.2 ± 0.1 ^b^	13.1 ± 0.1 ^c^	19.2 ± 0.5 ^e^	25.2 ± 0.4 ^f^	11.4 ± 0.3 ^a^	11.6 ± 0.3 ^a^	11.7 ± 0.1 ^a^	16.5 ± 0.2 ^d^
Ethyl palmitate (μg/L)	104.3 ± 1.0 ^f^	12.3 ± 0.3 ^d^	13.7 ± 0.2 ^e^	13.4 ± 0.1 ^e^	13.5 ± 0.1 ^e^	6.4 ± 0.1 ^a^	8.9 ± 0.1 ^b^	10.4 ± 0.2 ^c^	10.7 ± 0.1 ^c^
Ethyl linoleate (μg/L)	18.6 ± 0.4 ^b^	-	-	-	3.0 ± 0.1 ^a^	-	-	-	-
Ethyl oleate (μg/L)	10.0 ± 0.2 ^a^	-	-	-	-	-	-	-	-
Ethyl stearate (μg/L)	9.6 ± 0.4 ^a^	-	-	-	-	-	-	-	-
∑Volatile phenols (μg/L)	830.6 ± 10.6 ^h^	324.5 ± 7.1 ^a^	367.6 ± 1.8 ^b^	385.1 ± 1.8 ^d^	464.3 ± 6.3 ^g^	330.2 ± 5.1 ^a^	373.1 ± 0.4 ^c^	399.8 ± 0.6 ^e^	414.2 ± 5.4 ^f^
4-ethylphenol (μg/L)	111.0 ± 1.1 ^e^	-	4.2 ± 0.1 ^a^	4.5 ± 0.1 ^b^	5.8 ± 0.4 ^d^	4.0 ± 0.1 ^a^	4.7 ± 0.1 ^bc^	4.9 ± 0.2 ^c^	4.8 ± 0.1 ^c^
4-ethylguaiacol (μg/L)	139.7 ± 1.5 ^e^	5.1 ± 0.1 ^a^	7.7 ± 0.1 ^b^	7.6 ± 0.1 ^b^	9.2 ± 0.2 ^d^	5.1 ± 0.1 ^a^	7.2 ± 0.1 ^b^	7.5 ± 0.1 ^b^	8.4 ± 0.3 ^c^
2,4-Di-T-butylphenol (μg/L)	579.9 ± 8.1 ^h^	319.4 ± 7.0 ^a^	355.7 ± 1.6 ^b^	373.1 ± 1.7 ^d^	449.3 ± 5.8 ^g^	321.0 ± 5.0 ^a^	361.1 ± 0.2 ^c^	387.4 ± 0.3 ^e^	401.0 ± 5.0 ^f^

“-” not detected. Abbreviations: CW—initial conventional wine; CN–nanofiltration retentate of conventional wine; 1–2.5 MPa with cooling; 2–3.5 MPa with cooling; 3–4.5 MPa with cooling; 4–5.5 MPa with cooling; 5–2.5 MPa without cooling; 6–3.5 MPa without cooling; 7–4.5 MPa without cooling; 8–5.5 MPa without cooling.

**Table 4:** Volatile compounds identified in ecological Cabernet Sauvignon red wine and nanofiltration retentates at 2.5, 3.5, 4.5 and 5.5 MPa with cooling and without cooling. Different superscript letters (a, b, c, d, e, f, g, h, i) in the same row represent statistical difference by ANOVA, Fisher’s (LSD) test (*p* < 0.05).

Compound	EW	1EN	2EN	3EN	4EN	5EN	6EN	7EN	8EN
∑Acids (μg/L)	1634.4 ± 10.7 ^h^	189.5 ± 1.2 ^b^	255.4 ± 0.8 ^d^	270.0 ± 3.1 ^e^	319.8 ± 2.4 ^g^	177.1 ± 3.3 ^a^	226.8 ± 3.8 ^c^	253.7 ± 3.5 ^d^	289.7 ± 1.7 ^f^
Acetic acid (μg/L)	1043.0 ± 9.5 ^a^	-	-	-	-	-	-	-	-
Octanoic acid (μg/L)	311.9 ± 0.6 ^g^	62.9 ± 0.1 ^c^	67.9 ± 0.1 ^d^	59.3 ± 0.8 ^b^	75.4 ± 0.6 ^f^	55.8 ± 2.2 ^a^	55.0 ± 1.9 ^a^	61.3 ± 1.3 ^bc^	74.1 ± 0.3 ^e^
Decanoic acid (μg/L)	165.1 ± 0.4 ^h^	74.5 ± 0.4 ^b^	109.5 ± 0.2 ^d^	126.2 ± 1.2 ^f^	152.2 ± 0.8 ^g^	72.7 ± 0.3 ^a^	98.6 ± 0.9 ^c^	112.9 ± 0.9 ^e^	128.3 ± 1.1 ^f^
Lauric acid (μg/L)	83.9 ± 0.1 ^g^	38.3 ± 0.4 ^b^	60.8 ± 0.1 ^d^	64.7 ± 0.6 ^e^	69.0 ± 0.8 ^f^	36.8 ± 0.6 ^a^	59.1 ± 0.7 ^c^	63.3 ± 0.9 ^e^	68.2 ± 0.1 ^f^
Myristic acid (μg/L)	22.6 ± 0.2 ^g^	11.5 ± 0.2 ^b^	14.9 ± 0.3 ^d^	16.5 ± 0.4 ^e^	19.3 ± 0.1 ^f^	9.3 ± 0.2 ^a^	11.5 ± 0.2 ^b^	13.6 ± 0.3 ^c^	16.7 ± 0.1 ^e^
Palmitic acid (μg/L)	8.0 ± 0.1 ^c^	2.5 ± 0.1 ^a^	2.3 ± 0.2 ^a^	3.3 ± 0.1 ^b^	3.9 ± 0.2 ^b^	2.4 ± 0.1 ^a^	2.5 ± 0.1 ^a^	2.7 ± 0.1 ^a^	2.4 ± 0.01 ^a^
∑Alcohols (mg/L)	38.25 ± 0.48 ^h^	3.75 ± 0.03 ^c^	4.24 ± 0.04 ^d^	4.53 ± 0.02 ^e^	4.92 ± 0.10 ^g^	2.99 ± 0.02 ^a^	3.24 ± 0.04 ^b^	3.78 ± 0.03 ^c^	4.76 ± 0.02 ^f^
Isoamyl alcohol (mg/L)	31.79 ± 0.41 ^i^	2.33 ± 0.01 ^d^	2.71 ± 0.02 ^e^	2.92 ± 0.01 ^f^	3.21 ± 0.08 ^h^	1.71 ± 0.01 ^a^	1.81 ± 0.03 ^b^	2.16 ± 0.01 ^c^	3.07 ± 0.01 ^g^
2,3-butanediol (μg/L)	512.7 ± 0.8 ^f^	5.5 ± 0.3 ^a^	6.6 ± 0.4 ^b^	7.2 ± 0.1 ^b^	11.5 ± 0.2 ^e^	4.8 ± 0.4 ^a^	7.4 ± 0.4 ^b^	8.7 ± 0.1 ^c^	9.0 ± 0.2 ^d^
1-hexanol (μg/L)	755.2 ± 6.8 ^f^	54.5 ± 1.0 ^bc^	56.8 ± 0.6 ^d^	52.7 ± 1.1 ^b^	64.2 ± 1.1 ^e^	49.7 ± 1.1 ^a^	55.1 ± 0.7 ^c^	54.0 ± 0.4 ^bc^	56.3 ± 0.5 ^d^
Methionol (μg/L)	36.5 ± 0.5 ^a^	-	-	-	-	-	-	-	-
Benzyl alcohol (μg/L)	43.6 ± 0.6 ^a^	-	-	-	-	-	-	-	-
1-octanol (μg/L)	72.3 ± 0.3 ^i^	15.5 ± 0.2 ^c^	19.3 ± 0.3 ^e^	22.4 ± 0.3 ^g^	24.6 ± 0.3 ^h^	12.0 ± 0.1 ^a^	13.5 ± 0.4 ^b^	17.0 ± 0.1 ^d^	21.1 ± 0.4 ^f^
2-phenylethanol (mg/L)	4.93 ± 0.02 ^f^	1.29 ± 0.02 ^b^	1.39 ± 0.01 ^c^	1.45 ± 0.01 ^d^	1.52 ± 0.02 ^e^	1.17 ± 0.01 ^a^	1.28 ± 0.01 ^b^	1.47 ± 0.02 ^d^	1.52 ± 0.01 ^e^
Dodecanol (μg/L)	101.3 ± 0.4 ^g^	55.0 ± 2.8 ^b^	71.5 ± 0.1 ^d^	71.2 ± 0.4 ^d^	91.9 ± 1.6 ^f^	43.7 ± 1.0 ^a^	69.4 ± 0.2 ^c^	69.0 ± 0.4 ^c^	81.8 ± 0.6 ^e^
∑Carbonyl compounds (μg/L)	89.4 ± 0.5 ^g^	47.7 ± 0.7 ^b^	55.8 ± 1.2 ^d^	59.4 ± 1.0 ^e^	66.9 ± 1.0 ^f^	40.8 ± 0.9 ^a^	48.1 ± 0.9 ^b^	52.2 ± 1.2 ^c^	60.5 ± 0.9 ^e^
4-propylbenzaldehyde (μg/L)	25.0 ± 0.3 ^h^	11.0 ± 0.1 ^c^	11.8 ± 0.1 ^d^	14.2 ± 0.1 ^e^	16.4 ± 0.1 ^g^	9.4 ± 0.1 ^a^	9.8 ± 0.4 ^ab^	10.6 ± 0.5 ^bc^	15.1 ± 0.4 ^f^
Geranyl acetone (μg/L)	25.8 ± 0.1 ^g^	14.9 ± 0.3 ^c^	20.2 ± 0.6 ^e^	19.8 ± 0.3 ^e^	21.6 ± 0.3 ^f^	10.5 ± 0.3 ^a^	13.8 ± 0.1 ^b^	16.2 ± 0.5 ^d^	19.3 ± 0.2 ^e^
Lily aldehyde (μg/L)	18.3 ± 0.1 ^e^	10.8 ± 0.2 ^a^	11.5 ± 0.4 ^b^	12.5 ± 0.3 ^c^	13.6 ± 0.4 ^d^	10.5 ± 0.2 ^a^	12.1 ± 0.2 ^b^	12.8 ± 0.1 ^c^	12.7 ± 0.1 ^c^
Hexyl cinnamaldehyde (μg/L)	20.4 ± 0.1 ^e^	11.0 ± 0.2 ^a^	12.3 ± 0.1 ^b^	12.9 ± 0.3 ^bc^	15.3 ± 0.2 ^d^	10.5 ± 0.3 ^a^	12.5 ± 0.2 ^b^	12.6 ± 0.2 ^b^	13.3 ± 0.3 ^c^
∑Terpenes (μg/L)	210.9 ± 3.8 ^g^	73.1 ± 0.7 ^c^	76.0 ± 1.6 ^d^	81.7 ± 0.9 ^e^	85.7 ± 1.5 ^f^	57.4 ± 1.3 ^a^	68.2 ± 0.9 ^b^	74.6 ± 1.0 ^cd^	80.1 ± 1.2 ^e^
*α*-terpinolene (μg/L)	111.7 ± 1.8 ^g^	46.7 ± 0.2 ^e^	46.5 ± 0.5 ^e^	48.2 ± 0.2 ^f^	46.9 ± 0.5 ^e^	35.0 ± 0.8 ^a^	40.2 ± 0.1 ^b^	42.7 ± 0.1 ^c^	45.2 ± 0.5 ^d^
*β*-citronellol (μg/L)	17.7 ± 0.2 ^d^	5.3 ± 0.1 ^a^	5.9 ± 0.5 ^a^	8.3 ± 0.1 ^b^	9.6 ± 0.1 ^c^	5.2 ± 0.1 ^a^	5.3 ± 0.3 ^a^	8.6 ± 0.3 ^b^	9.8 ± 0.1 ^c^
*β*-damascenone (μg/L)	31.1 ± 0.6 ^f^	12.2 ± 0.1 ^c^	12.5 ± 0.2 ^c^	13.1 ± 0.3 ^d^	14.1 ± 0.3 ^e^	8.6 ± 0.1 ^a^	11.6 ± 0.3 ^b^	11.2 ± 0.2 ^b^	13.3 ± 0.3 ^d^
*β*-ionone (μg/L)	43.4 ± 1.2 ^e^	4.6 ± 0.3 ^a^	6.7 ± 0.1 ^b^	6.9 ± 0.3 ^bc^	8.8 ± 0.3 ^d^	4.1 ± 0.2 ^a^	6.6 ± 0.1 ^b^	6.5 ± 0.2 ^b^	7.2 ± 0.1 ^c^
Phenanthrene (μg/L)	7.0 ± 0.1 ^d^	4.3 ± 0.2 ^a^	4.4 ± 0.3 ^a^	5.2 ± 0.1 ^b^	6.3 ± 0.3 ^c^	4.6 ± 0.1 ^a^	4.6 ± 0.2 ^a^	5.6 ± 0.2 ^b^	4.5 ± 0.2 ^a^
∑Esters (mg/L)	4.12 ± 0.02 ^f^	2.24 ± 0.01 ^b^	2.40 ± 0.02 ^c^	2.60 ± 0.07 ^d^	2.87 ± 0.03 ^e^	1.93 ± 0.05 ^a^	2.30 ± 0.05 ^b^	2.43 ± 0.02 ^cd^	2.53 ± 0.08 ^d^
Ethyl hexanoate (μg/L)	141.5 ± 0.9 ^f^	41.0 ± 0.3 ^b^	44.7 ± 1.0 ^c^	50.9 ± 0.6 ^d^	60.6 ± 0.1 ^e^	37.8 ± 1.1 ^a^	38.9 ± 0.2 ^a^	43.8 ± 0.6 ^c^	51.7 ± 0.7 ^d^
Ethyl 4-hydroxybutanoate (μg/L)	33.4 ± 0.3 ^f^	13.8 ± 0.1 ^b^	15.7 ± 0.1 ^c^	16.4 ± 0.1 ^d^	17.2 ± 0.3 ^e^	12.7 ± 0.1 ^a^	14.3 ± 0.4 ^b^	15.7 ± 0.3 ^c^	15.7 ± 0.3 ^c^
Diethyl succinate (mg/L)	2.93 ± 0.01 ^f^	1.93 ± 0.01 ^b^	2.03 ± 0.01 ^c^	2.19 ± 0.06 ^d^	2.34 ± 0.02 ^e^	1.64 ± 0.04 ^a^	1.95 ± 0.04 ^b^	2.05 ± 0.01 ^c^	2.11 ± 0.06 ^cd^
Ethyl octanoate (μg/L)	367.8 ± 0.4 ^h^	103.7 ± 2.8 ^b^	125.9 ± 3.1 ^de^	122.2 ± 0.6 ^d^	155.2 ± 2.7 ^g^	93.9 ± 0.1 ^a^	119.6 ± 0.9 ^c^	130.2 ± 1.9 ^e^	145.9 ± 0.4 ^f^
Ethyl hydrogen succinate (μg/L)	248.6 ± 0.3 ^b^	-	-	-	43.5 ± 0.3 ^a^	-	-	-	-
Phenethyl acetate (μg/L)	69.6 ± 0.4 ^f^	26.5 ± 0.9 ^a^	33.2 ± 0.5 ^b^	57.0 ± 0.9 ^e^	53.2 ± 2.0 ^d^	28.2 ± 0.8 ^a^	34.3 ± 0.7 ^b^	40.8 ± 1.1 ^c^	52.4 ± 0.5 ^d^
Ethyl decanoate (μg/L)	19.5 ± 0.3 ^g^	12.0 ± 0.4 ^b^	16.6 ± 0.1 ^c^	19.4 ± 0.4 ^e^	25.0 ± 0.4 ^f^	10.0 ± 0.1 ^a^	16.3 ± 0.4 ^c^	16.7 ± 0.3 ^c^	18.1 ± 0.3 ^d^
Ethyl vanillate (μg/L)	30.0 ± 0.2 ^g^	11.9 ± 0.3 ^b^	14.9 ± 0.4 ^cd^	17.5 ± 0.1 ^e^	20.0 ± 0.3 ^f^	10.1 ± 0.2 ^a^	14.3 ± 0.2 ^c^	15.3 ± 0.5 ^d^	15.3 ± 0.1 ^d^
Ethyl laurate (μg/L)	40.3 ± 0.4 ^g^	23.3 ± 0.3 ^b^	28.9 ± 0.2 ^e^	29.1 ± 0.1 ^e^	35.3 ± 0.3 ^f^	20.6 ± 0.6 ^a^	25.1 ± 0.9 ^c^	27.6 ± 0.3 ^d^	28.7 ± 0.5 ^e^
Hexyl salicylate (μg/L)	15.4 ± 0.2 ^g^	6.6 ± 0.1 ^b^	8.7 ± 0.2 ^d^	9.3 ± 0.3 ^e^	14.1 ± 0.3 ^f^	5.0 ± 0.1 ^a^	7.7 ± 0.2 ^c^	9.3 ± 0.3 ^e^	13.4 ± 0.4 ^f^
Ethyl myristate (μg/L)	13.8 ± 0.2 ^f^	7.4 ± 0.1 ^e^	6.2 ± 0.1 ^d^	5.2 ± 0.1 ^c^	5.7 ± 0.2 ^c^	5.6 ± 0.4 ^c^	4.5 ± 0.1 ^b^	4.5 ± 0.2 ^b^	3.8 ± 0.1 ^a^
Diisobutyl phthalate (μg/L)	46.5 ± 0.2 ^h^	30.2 ± 0.5 ^b^	35.7 ± 0.4 ^d^	38.2 ± 0.6 ^e^	43.6 ± 1.2 ^g^	22.9 ± 0.1 ^a^	34.7 ± 0.4 ^c^	36.1 ± 0.3 ^d^	40.1 ± 0.7 ^f^
Ethyl pentadecanoate (μg/L)	13.6 ± 0.2 ^e^	6.3 ± 0.1 ^a^	7.5 ± 0.1 ^b^	7.9 ± 0.5 ^bc^	10.8 ± 0.1 ^d^	7.2 ± 0.2 ^b^	7.7 ± 0.3 ^b^	7.7 ± 0.3 ^b^	8.5 ± 0.1 ^c^
Methyl palmitate (μg/L)	14.5 ± 0.1 ^d^	11.2 ± 0.2 ^c^	10.3 ± 0.1 ^b^	8.8 ± 0.1 ^a^	8.8 ± 0.2 ^a^	11.6 ± 0.2 ^c^	10.5 ± 0.2 ^b^	9.4 ± 0.5 ^a^	9.0 ± 0.1 ^a^
Dibutyl phthalate (μg/L)	33.2 ± 0.2 ^f^	12.3 ± 0.2 ^b^	15.9 ± 0.1 ^d^	17.6 ± 0.5 ^e^	16.8 ± 0.3 ^e^	11.2 ± 0.1 ^a^	14.5 ± 0.1 ^c^	14.6 ± 0.3 ^c^	14.3 ± 0.1 ^c^
Ethyl palmitate (μg/L)	69.3 ± 1.1 ^e^	7.8 ± 0.2 ^a^	8.8 ± 0.2 ^b^	9.5 ± 0.1 ^c^	10.4 ± 0.1 ^d^	8.9 ± 0.4 ^b^	7.8 ± 0.2 ^a^	7.5 ± 0.1 ^a^	8.6 ± 0.3 ^b^
Ethyl linoleate (μg/L)	8.9 ± 0.1 ^b^	-	-	-	2.8 ± 0.1 ^a^	-	-	-	-
Ethyl oleate (μg/L)	9.5 ± 0.2 ^a^	-	-	-	-	-	-	-	-
Ethyl stearate (μg/L)	9.5 ± 0.5 ^a^	-	-	-	-	-	-	-	-
∑Volatile phenols (μg/L)	811.8 ± 5.2 ^g^	366.8 ± 3.1 ^b^	467.5 ± 2.5 ^e^	469.4 ± 1.4 ^e^	495.2 ± 3.1 ^f^	305.9 ± 4.1 ^a^	380.0 ± 0.9 ^c^	433.8 ± 1.6 ^d^	471.4 ± 2.4 ^e^
4-ethylphenol (μg/L)	127.7 ± 0.9 ^d^	-	5.1 ± 0.1 ^c^	5.1 ± 0.1 ^c^	5.2 ± 0.2 ^c^	4.0 ± 0.1 ^a^	4.8 ± 0.1 ^b^	4.5 ± 0.3 ^b^	4.8 ± 0.1 ^b^
4-ethylguaiacol (μg/L)	142.1 ± 0.2 ^d^	4.8 ± 0.1 ^a^	5.7 ± 0.1 ^c^	5.7 ± 0.1 ^c^	5.7 ± 0.1 ^c^	4.6 ± 0.2 ^a^	5.2 ± 0.1 ^b^	5.7 ± 0.1 ^c^	5.8 ± 0.1 ^c^
2,4-Di-T-butylphenol (μg/L)	542.0 ± 4.1 ^g^	361.9 ± 3.0 ^b^	456.6 ± 2.3 ^e^	458.6 ± 1.2 ^e^	484.3 ± 2.8 ^f^	297.3 ± 3.8 ^a^	370.0 ± 0.7 ^c^	423.5 ± 1.2 ^d^	460.8 ± 2.3 ^e^

“-” not detected. Abbreviations: EW–initial ecological wine; EN—nanofiltration retentate of ecological wine; 1–2.5 MPa with cooling; 2–3.5 MPa with cooling; 3–4.5 MPa with cooling; 4–5.5 MPa with cooling; 5–2.5 MPa without cooling; 6–3.5 MPa without cooling; 7–4.5 MPa without cooling; 8–5.5 MPa without cooling.

**Table 5:** Chemical composition of initial conventional Cabernet Sauvignon wine and nanofiltration retentates at 2.5, 3.5, 4.5 and 5.5 MPa with cooling and without cooling. Different superscript letters (a, b, c, d, e, f, g) in the same column represent statistically different values (*p* < 0.05; ANOVA, Fisher’s (LSD) test).

Sample	Ethanol (vol.%)	Glycerol (g/L)	Density (g/L)	Free SO_2_ (mg/L)	Total SO_2_ (mg/L)	Reducing Sugars (g/L)	CO_2_ (g/L)
CW	13.74 ± 0.01 ^e^	9.7 ± 0.1 ^c^	0.9946 ± 0.0003 ^a^	12.80 ± 0.1 ^c^	43.52 ± 0.01 ^b^	4.1 ± 0.1 ^b^	232.61 ± 0.12 ^g^
1CN	5.65 ± 0.01 ^a^	4.8 ± 0.2 ^a^	1.0024 ± 0.0003 ^b^	11.52 ± 0.1 ^b^	46.08 ± 0.02 ^d^	3.0 ± 0.2 ^a^	206.18 ± 0.11 ^f^
2CN	5.87 ± 0.04 ^b^	5.1 ± 0.1 ^a^	1.0026 ± 0.0002 ^b^	11.52 ± 0.1 ^b^	48.64 ± 0.03 ^e^	3.2 ± 0.1 ^a^	195.23 ± 0.09 ^e^
3CN	5.77 ± 0.06 ^b^	5.1 ± 0.1 ^a^	1.0026 ± 0.0001 ^b^	10.24 ± 0.1 ^a^	44.80 ± 0.03 ^c^	3.2 ± 0.1 ^a^	171.28 ± 0.21 ^d^
4CN	6.16 ± 0.07 ^c^	5.8 ± 0.1 ^b^	1.0030 ± 0.0003 ^b^	10.24 ± 0.1 ^a^	44.80 ± 0.04 ^c^	3.4 ± 0.3 ^a^	148.59 ± 0.17 ^c^
5CN	5.66 ± 0.02 ^a^	4.9 ± 0.1 ^a^	1.0027 ± 0.0002 ^b^	11.52 ± 0.1 ^b^	46.08 ± 0.02 ^d^	3.3 ± 0.2 ^a^	145.66 ± 0.24 ^b^
6CN	5.81 ± 0.02 ^b^	5.1 ± 0.1 ^a^	1.0029 ± 0.0001 ^b^	11.52 ± 0.1 ^b^	46.08 ± 0.01 ^d^	3.3 ± 0.1 ^a^	145.89 ± 0.16 ^b^
7CN	5.94 ± 0.01 ^c^	5.1 ± 0.2 ^a^	1.0030 ± 0.0003 ^b^	10.24 ± 0.1 ^a^	42.24 ± 0.01 ^a^	3.4 ± 0.2 ^a^	143.90 ± 0.35 ^a^
8CN	6.30 ± 0.07 ^d^	5.9 ± 0.1 ^b^	1.0030 ± 0.0003 ^b^	10.24 ± 0.1 ^a^	42.24 ± 0.01 ^a^	3.3 ± 0.2 ^a^	143.25 ± 0.33 ^a^

Abbreviations: CW—initial conventional wine; CN—nanofiltration retentate of conventional wine; 1–2.5 MPa with cooling; 2–3.5 MPa with cooling; 3–4.5 MPa with cooling; 4–5.5 MPa with cooling; 5–2.5 MPa without cooling; 6–3.5 MPa without cooling; 7–4.5 MPa without cooling; 8–5.5 MPa without cooling.

**Table 6:** Chemical composition of initial ecological Cabernet Sauvignon wine and nanofiltration retentates at 2.5, 3.5, 4.5 and 5.5 MPa with cooling and without cooling. Different superscript letters (a, b, c, d, e, f) in the same column represent statistically different values (*p* < 0.05; ANOVA, Fisher’s (LSD) test).

Sample	Ethanol (vol.%)	Glycerol (g/L)	Density (g/L)	Free SO_2_ (mg/L)	Total SO_2_ (mg/L)	Reducing Sugars (g/L)	CO_2_ (g/L)
EW	13.53 ± 0.02 ^e^	9.3 ± 0.2 ^c^	0.9946 ± 0.0002 ^a^	12.80 ± 0.1 ^c^	43.52 ± 0.01 ^a^	4.1 ± 0.1 ^a^	444.64 ± 0.22 ^f^
1EN	5.63 ± 0.01 ^b^	4.7 ± 0.1 ^b^	1.0028 ± 0.0001 ^b^	11.52 ± 0.1 ^b^	47.36 ± 0.02 ^c^	3.9 ± 0.1 ^a^	160.15 ± 0.13 ^e^
2EN	5.89 ± 0.09 ^c^	5.2 ± 0.1 ^c^	1.0026 ± 0.0003 ^b^	11.52 ± 0.1 ^b^	49.92 ± 0.03 ^d^	3.9 ± 0.2 ^a^	148.59 ± 0.11 ^d^
3EN	5.66 ± 0.14 ^c^	5.0 ± 0.2 ^c^	1.0026 ± 0.0003 ^b^	11.52 ± 0.1 ^a^	49.92 ± 0.03 ^d^	3.8 ± 0.2 ^a^	147.23 ± 0.22 ^c^
4EN	5.95 ± 0.06 ^d^	5.1 ± 0.3 ^cd^	1.0030 ± 0.0003 ^b^	10.24 ± 0.1 ^a^	49.92 ± 0.04 ^d^	3.8 ± 0.2 ^a^	147.10 ± 0.25 ^c^
5EN	5.11 ± 0.04 ^a^	4.0 ± 0.3 ^a^	1.0027 ± 0.0002 ^b^	10.24 ± 0.1 ^b^	46.08 ± 0.02 ^b^	4.0 ± 0.1 ^a^	144.06 ± 0.09 ^b^
6EN	5.58 ± 0.05 ^b^	4.7 ± 0.2 ^b^	1.0026 ± 0.0002 ^b^	10.24 ± 0.1 ^b^	46.08 ± 0.01 ^b^	3.8 ± 0.2 ^a^	142.68 ± 0.13 ^a^
7EN	5.76 ± 0.05 ^c^	5.1 ± 0.1 ^c^	1.0031 ± 0.0003 ^b^	10.24 ± 0.1 ^a^	46.08 ± 0.01 ^b^	4.1 ± 0.1 ^a^	142.68 ± 0.24 ^a^
8EN	6.10 ± 0.08 ^d^	5.6 ± 0.2 ^d^	1.0030 ± 0.0002 ^b^	10.24 ± 0.1 ^a^	43.52 ± 0.01 ^a^	4.0 ± 0.2 ^a^	142.68 ± 0.17 ^a^

Abbreviations: EW—initial ecological wine; EN—nanofiltration retentate of ecological wine; 1–2.5 MPa with cooling; 2–3.5 MPa with cooling; 3–4.5 MPa with cooling; 4–5.5 MPa with cooling; 5–2.5 MPa without cooling; 6–3.5 MPa without cooling; 7–4.5 MPa without cooling; 8–5.5 MPa without cooling.

**Table 7:** Total acidity, volatile acidity, malic, lactic, citric, sorbic and tartaric acid and pH in initial conventional Cabernet Sauvignon wine and nanofiltration retentates at 2.5, 3.5, 4.5 and 5.5 MPa with cooling and without cooling. Different superscript letters (a, b, c, d, e, f, g, h, i) in the same column represent statistically different values (*p* < 0.05; ANOVA, Fisher’s (LSD) test).

Sample	Total Acidity (g/L)	Volatile Acidity (g/L)	Malic Acid (g/L)	Lactic Acid (g/L)	Citric Acid (g/L)	Sorbic Acid (g/L)	Tartaric Acid (g/L)	pH
CW	4.9 ± 0.1 ^b^	0.9 ± 0.1 ^b^	0.8 ± 0.1 ^b^	2.1 ± 0.1 ^b^	0.29 ± 0.01 ^b^	132.0 ± 0.1 ^i^	0.7 ± 0.2 ^a^	3.92 ± 0.02 ^c^
1CN	3.1 ± 0.3 ^a^	0.4 ± 0.1 ^a^	0.4 ± 0.2 ^a^	1.0 ± 0.2 ^a^	0.19 ± 0.02 ^a^	2.0 ± 0.1 ^a^	0.7 ± 0.1 ^a^	3.70 ± 0.01 ^a^
2CN	3.4 ± 0.1 ^a^	0.4 ± 0.1 ^a^	0.3 ± 0.1 ^a^	1.1 ± 0.1 ^a^	0.17 ± 0.01 ^a^	8.0 ± 0.1 ^d^	0.8 ± 0.2 ^a^	3.72 ± 0.01 ^a^
3CN	3.4 ± 0.1 ^a^	0.4 ± 0.1 ^a^	0.3 ± 0.1 ^a^	1.1 ± 0.1 ^a^	0.18 ± 0.02 ^a^	7.0 ± 0.1 ^c^	0.8 ± 0.2 ^a^	3.71 ± 0.01 ^a^
4CN	3.6 ± 0.3 ^a^	0.5 ± 0.1 ^a^	0.3 ± 0.1 ^a^	1.2 ± 0.2 ^a^	0.15 ± 0.03 ^a^	22.0 ± 0.1 ^g^	0.9 ± 0.2 ^a^	3.74 ± 0.01 ^ab^
5CN	3.4 ± 0.1 ^a^	0.4 ± 0.1 ^a^	0.3 ± 0.2 ^a^	1.0 ± 0.1 ^a^	0.15 ± 0.02 ^a^	17.0 ± 0.1 ^f^	0.9 ± 0.2 ^a^	3.71 ± 0.01 ^a^
6CN	3.4 ± 0.1 ^a^	0.4 ± 0.1 ^a^	0.3 ± 0.2 ^a^	1.1 ± 0.1 ^a^	0.17 ± 0.01 ^a^	14.0 ± 0.1 ^e^	0.8 ± 0.1 ^a^	3.72 ± 0.01 ^a^
7CN	3.3 ± 0.2 ^a^	0.4 ± 0.1 ^a^	0.3 ± 0.1 ^a^	1.0 ± 0.1 ^a^	0.18 ± 0.01 ^a^	6.0 ± 0.1 ^b^	0.7 ± 0.2 ^a^	3.72 ± 0.01 ^a^
8CN	3.5 ± 0.3 ^a^	0.5 ± 0.1 ^a^	0.3 ± 0.1 ^a^	1.3 ± 0.3 ^a^	0.14 ± 0.03 ^a^	32.0 ± 0.1 ^h^	0.6 ± 0.2 ^a^	3.76 ± 0.01 ^b^

Abbreviations: CW—initial conventional wine; CN—nanofiltration retentate of conventional wine; 1–2.5 MPa with cooling; 2–3.5 MPa with cooling; 3–4.5 MPa with cooling; 4–5.5 MPa with cooling; 5–2.5 MPa without cooling; 6–3.5 MPa without cooling; 7–4.5 MPa without cooling; 8–5.5 MPa without cooling.

**Table 8:** Total acidity, volatile acidity, malic, lactic, citric, sorbic and tartaric acid and pH in initial ecological Cabernet Sauvignon wine and nanofiltration retentates at 2.5, 3.5, 4.5 and 5.5 MPa with cooling and without cooling. Different superscript letters (a, b, c) in the same column represent statistically different values (*p* < 0.05; ANOVA, Fisher’s (LSD) test).

Sample	Total Acidity (g/L)	Volatile Acidity (g/L)	Malic Acid (g/L)	Lactic Acid (g/L)	Citric Acid (g/L)	Sorbic Acid (g/L)	Tartaric Acid (g/L)	pH
EW	5.1 ± 0.1 ^b^	0.9 ± 0.1 ^b^	0.6 ± 0.1 ^b^	1.8 ± 0.1 ^b^	0.31 ± 0.01 ^b^	47.0± 0.1 ^a^	0.7 ± 0.1 ^a^	3.75 ± 0.01 ^c^
1EN	3.3 ± 0.1 ^a^	0.4 ± 0.1 ^a^	0.2 ± 0.1 ^a^	0.8 ± 0.1 ^a^	0.22 ± 0.01 ^a^	-	0.8 ± 0.2 ^a^	3.65 ± 0.01 ^b^
2EN	3.6 ± 0.3 ^a^	0.4 ± 0.1 ^a^	0.2 ± 0.1 ^a^	0.9 ± 0.1 ^a^	0.20 ± 0.02 ^a^	-	0.8 ± 0.1 ^a^	3.65 ± 0.01 ^b^
3EN	3.4 ± 0.1 ^a^	0.4 ± 0.1 ^a^	0.2 ± 0.1 ^a^	0.8 ± 0.1 ^a^	0.23 ± 0.02 ^a^	-	0.7 ± 0.2 ^a^	3.63 ± 0.01 ^b^
4EN	3.5 ± 0.2 ^a^	0.4 ± 0.1 ^a^	0.2 ± 0.1 ^a^	0.9 ± 0.1 ^a^	0.22 ± 0.01 ^a^	-	0.8 ± 0.1 ^a^	3.63 ± 0.01 ^b^
5EN	3.1 ± 0.3 ^a^	0.3 ± 0.1 ^a^	0.3 ± 0.1 ^a^	0.6 ± 0.3 ^a^	0.24 ± 0.02 ^a^	-	0.7 ± 0.2 ^a^	3.60 ± 0.01 ^a^
6EN	3.3 ± 0.1 ^a^	0.4 ± 0.1 ^a^	0.2 ± 0.1 ^a^	0.7 ± 0.2 ^a^	0.22 ± 0.01 ^a^	-	0.7 ± 0.2 ^a^	3.62 ± 0.01 ^a^
7EN	3.5 ± 0.2 ^a^	0.4 ± 0.1 ^a^	0.2 ± 0.1 ^a^	0.8 ± 0.2 ^a^	0.20 ± 0.03 ^a^	-	0.8 ± 0.1 ^a^	3.64 ± 0.01 ^ab^
8EN	3.6 ± 0.3 ^a^	0.4 ± 0.1 ^a^	0.1 ± 0.1 ^a^	0.9 ± 0.1 ^a^	0.19 ± 0.03 ^a^	-	0.8 ± 0.1 ^a^	3.66 ± 0.01 ^b^

Abbreviations: EW—initial ecological wine; EN—nanofiltration retentate of ecological wine; 1–2.5 MPa with cooling; 2–3.5 MPa with cooling; 3–4.5 MPa with cooling; 4–5.5 MPa with cooling; 5–2.5 MPa without cooling; 6–3.5 MPa without cooling; 7–4.5 MPa without cooling; 8–5.5 MPa without cooling.

**Table 9:** Content of elements in the initial conventional Cabernet Sauvignon wine and NF retentates obtained at 2.5, 3.5, 4.5 and 5.5 MPa with cooling and without cooling. Significant differences (*p* < 0.05) between samples are indicated by different superscript letters (a, b, c, d, e) within the row (ANOVA, Fisher’s LSD test).

Element	CW	1CN	2CN	3CN	4CN	5CN	6CN	7CN	8CN
K (mg/L)	597.7 ± 55.9 ^c^	509.0 ± 8.2 ^a^	499.0 ± 6.8 ^a^	627.4 ± 12.5 ^c^	672.7 ± 19.3 ^c^	505.3 ± 25.3 ^ab^	548.2 ± 12.5 ^b^	526.5 ± 29.3 ^ab^	517.3 ± 27.1 ^ab^
Ca (mg/L)	55.7 ± 3.2 ^c^	40.6 ± 1.0 ^a^	51.1 ± 2.2 ^bc^	49.8 ± 1.4 ^b^	54.0 ± 2.0 ^c^	41.6 ± 2.0 ^a^	51.8 ± 1.3 ^bc^	52.9 ± 1.8 ^bc^	51.9 ± 3.4 ^bc^
Mn (μg/L)	1925.6 ± 33.8 ^d^	1565.6 ± 15.0 ^b^	1681.7 ± 43.9 ^c^	1918.8 ± 69.3 ^d^	1984.0 ± 50.5 ^d^	1478.1 ± 37.7 ^a^	1502.2± 15.6 ^a^	1507.0 ± 36.8 ^a^	1593.0 ± 15.4 ^b^
Fe (μg/L)	1785.0± 38.6 ^c^	1355.0 ± 45.9 ^a^	1351.0 ± 40.4 ^a^	1645.0 ± 23.0 ^b^	1835.0 ± 57.0 ^c^	1425.0 ± 31.4 ^a^	1397.9 ± 20.1 ^a^	1429.0 ± 36.2 ^a^	1419.6 ± 59.5 ^a^
Cu (μg/L)	447.9 ± 21.4 ^b^	442.6 ± 34.1 ^b^	452.0 ± 11.2 ^b^	454.0 ± 12.4 ^b^	465.8 ± 37.9 ^b^	329.2 ± 36.6 ^a^	355.5 ± 36.2 ^a^	352.9 ± 32.5 ^a^	331.5 ± 34.4 ^a^
Zn (μg/L)	1400.5 ± 14.8 ^d^	1409.2 ± 33.0 ^d^	1268.8 ± 27.4 ^c^	1230.4 ± 7.4 ^c^	1195.0 ± 40.4 ^bc^	1380.0 ± 27.0 ^d^	1226.8 ± 24.8 ^c^	1130.2 ± 20.4 ^b^	986.6 ± 34.8 ^a^
Br (μg/L)	21.8 ± 1.1 ^e^	11.4 ± 0.4 ^a^	13.3 ± 0.7 ^b^	17.1 ± 0.6 ^c^	18.4 ± 0.2 ^d^	11.2 ± 0.6 ^a^	10.9 ± 0.7 ^a^	13.3 ± 1.9 ^ab^	13.6 ± 0.8 ^b^
Rb (μg/L)	1062.9 ± 48.4 ^c^	801.3 ± 32.0 ^a^	867.1 ± 63.0 ^ab^	1042.9 ± 57.0 ^c^	1015.7 ± 43.0 ^c^	822.0 ± 24.6 ^a^	903.3 ± 36.0 ^b^	934.3 ± 35.0 ^b^	912.0 ± 14.2 ^b^
Sr (μg/L)	260.6 ± 9.9 ^c^	208.4 ± 7.0 ^a^	235.7 ± 9.7 ^b^	264.1 ± 14.1 ^c^	267.7 ± 19.0 ^c^	216.5 ± 14.0 ^ab^	233.8 ± 4.2 ^b^	236.3 ± 2.0 ^b^	220.7 ± 8.0 ^b^
Pb (μg/L)	20.7 ± 2.5 ^c^	11.5 ± 1.5 ^a^	12.5 ± 2.9 ^a^	11.4 ± 1.6 ^a^	13.6 ± 1.6 ^ab^	14.0 ± 1.5 ^ab^	13.3 ± 1.3 ^a^	14.5 ± 1.2 ^ab^	13.6 ± 1.6 ^a^

Abbreviations: CW—initial conventional wine; CN—nanofiltration retentate of conventional wine; 1–2.5 MPa with cooling; 2–3.5 MPa with cooling; 3–4.5 MPa with cooling; 4–5.5 MPa with cooling; 5–2.5 MPa without cooling; 6–3.5 MPa without cooling; 7–4.5 MPa without cooling; 8–5.5 MPa without cooling.

**Table 10:** Content of elements in the initial ecological Cabernet Sauvignon wine and NF retentates obtained at 2.5, 3.5, 4.5 and 5.5 MPa with cooling and without cooling. Significant differences (*p* < 0.05) between samples are indicated by different superscript letters (a, b, c, d, e) within the row (ANOVA, Fisher’s LSD test).

Element	EW	1EN	2EN	3EN	4EN	5EN	6EN	7EN	8EN
K (mg/L)	748.7 ± 28.9 ^b^	435.8 ± 19.7 ^a^	449.7 ± 21.2 ^a^	447.6 ± 11.1 ^a^	445.9 ± 15.8 ^a^	446.5 ± 18.8 ^a^	446.8 ± 18.2 ^a^	426.7 ± 13.6 ^a^	428.6 ± 17.6 ^a^
Ca (mg/L)	50.7 ± 0.1 ^b^	42.0 ± 3.9 ^a^	42.5 ± 5.9 ^a^	42.8 ± 3.5 ^a^	40.7 ± 4.4 ^a^	44.8 ± 1.6 ^a^	41.2 ± 3.3 ^a^	41.7 ± 1.9 ^a^	37.1 ± 6.9 ^ab^
Mn (μg/L)	1838.2 ± 0.1 ^d^	1309.8 ± 8.2 ^a^	1438.4 ± 6.8 ^c^	1469.2 ± 22.5 ^c^	1426.5 ± 19.3 ^c^	1425.3 ± 25.3 ^c^	1365.4 ± 12.5 ^b^	1421.2 ± 29.3 ^c^	1294.9 ± 27.1 ^a^
Fe (μg/L)	1317.8 ± 47.7 ^d^	1180.5 ± 53.2 ^a^	1210.8 ± 48.0 ^ab^	1231.9 ± 12.7 ^b^	1288.8 ± 17.4 ^c^	1216.1 ± 22.6 ^a^	1247.5 ± 14.0 ^ab^	1215.7 ± 35.5 ^a^	1180.3 ± 21.5 ^a^
Cu (μg/L)	496.8 ± 24.6 ^d^	286.2 ± 14.9 ^bc^	296.4 ± 1.8 ^b^	323.2 ± 19.2 ^c^	324.2 ± 17.2 ^c^	257.9 ± 20.6 ^ab^	249.9 ± 1.3 ^a^	286.9 ± 27.2 ^bc^	313.3 ± 10.0 ^c^
Zn (μg/L)	1212.9 ± 71.0 ^e^	948.0 ± 20.7 ^c^	888.8 ± 13.6 ^b^	867.1 ± 19.3 ^b^	824.0 ± 56.3 ^ab^	1113.4 ± 58.9 ^d^	917.5 ± 34.2 ^bc^	853.7 ± 29.3 ^ab^	815.5 ± 25.6 ^a^
Br (μg/L)	24.9 ± 2.3 ^d^	16.7 ± 2.2 ^a^	20.4 ± 1.9 ^ab^	25.7 ± 1.5 ^c^	25.9 ± 1.8 ^c^	17.7 ± 1.5 ^a^	16.8 ± 1.9 ^a^	23.4 ± 0.9 ^bc^	19.5 ± 1.9 ^a^
Rb (μg/L)	1663.1 ± 10.2 ^c^	1178.4 ± 64.9 ^a^	1255.1 ± 39.6 ^a^	1412.2 ± 20.1 ^b^	1361.4 ± 40.6 ^b^	1278.4 ± 28.1 ^a^	1224.9 ± 44.3 ^a^	1269.5 ± 37.2 ^a^	1234.7 ± 46.7 ^a^
Sr (μg/L)	520.6 ± 49.1 ^d^	365.1 ± 15.0 ^ab^	386.3 ± 13.9 ^b^	457.0 ± 30.3 ^c^	386.9 ± 7.7 ^b^	377.9 ± 17.4 ^ab^	344.6 ± 18.2 ^a^	420.8 ± 19.1 ^c^	356.6 ± 17.8 ^a^
Pb (μg/L)	25.8 ± 1.1 ^c^	22.1 ± 1.1 ^b^	27.4 ± 2.1 ^c^	27.2 ± 2.1 ^c^	25.3 ± 1.8 ^c^	14.9 ± 2.0 ^a^	15.3 ± 2.0 ^a^	22.6 ± 1.5 ^b^	23.4 ± 2.2 ^bc^

Abbreviations: EW—initial ecological wine; EN—nanofiltration retentate of ecological wine; 1–2.5 MPa with cooling; 2–3.5 MPa with cooling; 3–4.5 MPa with cooling; 4–5.5 MPa with cooling; 5–2.5 MPa without cooling; 6–3.5 MPa without cooling; 7–4.5 MPa without cooling; 8–5.5 MPa without cooling.

## Data Availability

Not available.
